# Patient-Derived Organoids Predict Treatment Response in Metastatic Colorectal Cancer

**DOI:** 10.1158/1078-0432.CCR-25-1564

**Published:** 2025-09-22

**Authors:** Lidwien P. Smabers, G. Emerens Wensink, Carla S. Verissimo, Mayke Doorn, Tao Yang, Timo Voskuilen, Maarten A. Huismans, Liselot B.J. Valkenburg-van Iersel, Geert A. Cirkel, Elske C. Gootjes, Henk M.W. Verheul, Frank J. Jeurissen, Guus M. Bol, Hilde H. Nienhuis, Manon N.G.J.A. Braat, Edwin Cuppen, Robert G.J. Vries, Frederieke H. van der Baan, Sjoerd G. Elias, Onno Kranenburg, Miriam Koopman, Sylvia F. Boj, Jeanine M.L. Roodhart

**Affiliations:** 1 Department of Medical Oncology, University Medical Center Utrecht (UMCU), Utrecht University, Utrecht, the Netherlands.; 2 Division of Imaging and Cancer, Laboratory of Translational Oncology, UMCU, Utrecht University, Utrecht, the Netherlands.; 3 HUB Organoids B.V., Utrecht, the Netherlands, Part of the Life Science Business of Merck KGaA, Darmstadt, Germany.; 4 Department of Molecular Cancer Research, Center for Molecular Medicine, UMCU, Utrecht University, Utrecht, the Netherlands.; 5 Department of Internal Medicine, Division of Medical Oncology, GROW, Maastricht University Medical Center, Maastricht, the Netherlands.; 6 Department of Medical Oncology, Meander Medical Center, Amersfoort, the Netherlands.; 7 Department of Medical Oncology, Radboud University Medical Center, Nijmegen, the Netherlands.; 8 Department of Internal Medicine, Haaglanden Medical Center, The Hague, the Netherlands.; 9 UCSF Helen Diller Family Comprehensive Cancer Center, San Francisco, California.; 10 Hartwig Medical Foundation, Amsterdam, the Netherlands.; 11 Department of Radiology, UMCU, Utrecht University, Utrecht, the Netherlands.; 12 Department of Epidemiology, Julius Center for Health Sciences and Primary Care, UMCU, Utrecht University, Utrecht, the Netherlands.

## Abstract

**Purpose:**

Accurately predicting treatment response in metastatic colorectal cancer (mCRC) is critical to avoid unnecessary toxicity and improve patient outcomes. Patient-derived organoids (PDO) are promising models, but larger prospective studies are needed to confirm their predictive value.

**Experimental Design:**

Patients with mCRC underwent a metastatic biopsy for PDO establishment, before starting new systemic treatment. Predictors of PDO establishment were identified. PDOs were incubated with a seven-drug panel, including the patient’s treatment, to determine drug sensitivity as measured by CyQUANT cell viability [area under the nonfitted “curve” of the raw viability or growth rate inhibition values (AUC and GR_AUC_) and concentration that gives half-maximal viability or GR inhibition (IC_50 _and GR_50_)]. Patient response was measured by size change of biopsied and all target lesions. The diagnostic performance was evaluated by positive predictive value, negative predictive value, and area under the ROC curve. Additionally, the association between PDO response and survival was assessed.

**Results:**

A total of 232 patients were included, and 205 biopsies were obtained. PDO establishment success increased from 22% to 75%, yielding 52% overall. Male sex, increased lactate dehydrogenase, biopsy in academic hospitals, optimized culture conditions, and experience were related to PDO establishment success. In this interim analysis, focused on oxaliplatin-based doublet chemotherapy, 42 PDOs were screened. PDO drug sensitivity significantly correlated with the response of the biopsied lesion (*R* = 0.41–0.49, *P* < 0.011) and all target lesions (*R* = 0.54–0.60, *P* < 0.001) for all treatments combined. The 5-fluorouracil and oxaliplatin PDO screens demonstrated high predictive accuracy (positive predictive value: 0.78; negative predictive value: 0.80; area under the ROC curve: 0.78–0.88) and were associated with progression-free survival and overall survival (*P* = 0.016 and 0.049).

**Conclusions:**

We identified predictors for successful mCRC PDO establishment and validated that PDOs can accurately predict patient outcomes during systemic treatment, specifically with 5-fluorouracil and oxaliplatin.

**Translational Relevance**

The OPTIC trial is a multicenter prospective study to validate that patient-derived organoid (PDO) response correlates with patient response to standard systemic metastatic colorectal cancer treatment. We show that PDOs can predict both radiological tumor response and survival, particularly for oxaliplatin-based chemotherapy. The high predictive accuracy highlights their value for the identification of (in) effective therapies, supporting their use in personalized cancer treatment. PDO-based personalized medicine in clinical practice enables tailored treatment selection, enhancing patient outcomes and minimizing exposure to ineffective therapies. The discovery that both patient characteristics and laboratory conditions critically influence PDO establishment paves the way for optimizing PDO establishment rates, enabling a broader application. The successful translation of PDO-based drug response into patient outcome prediction marks an important advance toward establishing PDO-guided therapy for patients with metastatic colorectal cancer.

## Introduction

Despite advancements in systemic therapies for metastatic colorectal cancer (mCRC), treatment efficacy remains suboptimal. Currently, the doublet or triplet chemotherapy regimen fluorouracil/capecitabine combined with oxaliplatin (FOLFOX or CAPOX, with EGFR or VEGF inhibition) and/or irinotecan (FOLFOXIRI or FOLFIRI, with EGFR or VEGF inhibition) is recommended as first-line treatment (1, 2). Large randomized clinical trials demonstrate that approximately 20% to 40% of patients do not benefit from standard first-line doublet chemotherapy regimens (3, 4). Although genomic biomarkers such as *BRAF* and *KRAS* are used to guide therapy selection, the availability of genomic biomarkers to guide chemotherapy selection is limited (5). To tailor treatments more effectively and prevent unnecessary exposure to ineffective chemotherapies, innovative biomarkers are needed to guide therapeutic decision-making.

Since the discovery in 2009, organoid culture technology has enabled the unlimited expansion of adult stem cells, which can subsequently be used for functional assays (6, 7). Importantly, patient-derived organoids (PDO) maintain the phenotypical and genomic features of the original tumor (8–10). Organoid technology allows the expansion of both healthy and tumor tissue, providing an array of opportunities to improve health care. One of these opportunities is *in vitro* drug sensitivity screening to predict therapy response. Several retrospective studies have shown that PDO response matches the patient response to systemic therapies. In pooled analyses, the specificity and sensitivity of colorectal cancer PDO-based predictive scores exceeded 70% (11, 12). However, these studies included small numbers of patients and were performed in favorable settings with large tumor specimens instead of needle biopsies and no predefined cutoff for sensitivity scores. This may have led to an overly optimistic view of the value and feasibility of PDO technology in routine practice.

The process of culturing PDOs has evolved significantly in the past decade. Culture media have been refined and optimized to improve PDO growth and function. Challenges such as optimizing culture conditions, variations in tumor cell purity, and the potential influence of patient and tumor characteristics could hinder successful PDO culture (13–15). In this study, we aim to identify factors that influence the successful cultivation of PDOs. Gaining more knowledge of these factors will improve the application of PDO models, which is crucial for their adoption in clinical care.

For mCRC, larger prospective studies are needed to confirm the predictive value of standardized PDO screens. OPTIC is a multicenter prospective study that addresses this critical gap by validating the potential value of incorporating organoid technology in the stratification of patients for treatment with antineoplastic agents. The study aims to validate that *in vitro* PDO response correlates with *in vivo* response to standard treatment in patients with mCRC. The primary clinical endpoint was the response of the index metastasis to standard-of-care treatment, which is biopsied for PDO culture, measured by change in size on a CT scan. Secondary clinical endpoints were response according to RECIST 1.1, response of all metastatic target lesions, and dichotomized response. This interim analysis focuses on a common first-line treatment: 5-fluorouracil (5-FU) and oxaliplatin. In early-line treatments, tumors are less affected by prior therapies, enabling clearer predictions of treatment outcomes. Moreover, the first-line setting has the widest range of therapeutic options, underscoring the relevance of efficacy prediction. This might help clinicians identify the most effective treatment for each patient, marking a significant step toward personalized anticancer therapy.

## Materials and Methods

### Study cohort

**Figure 1.**
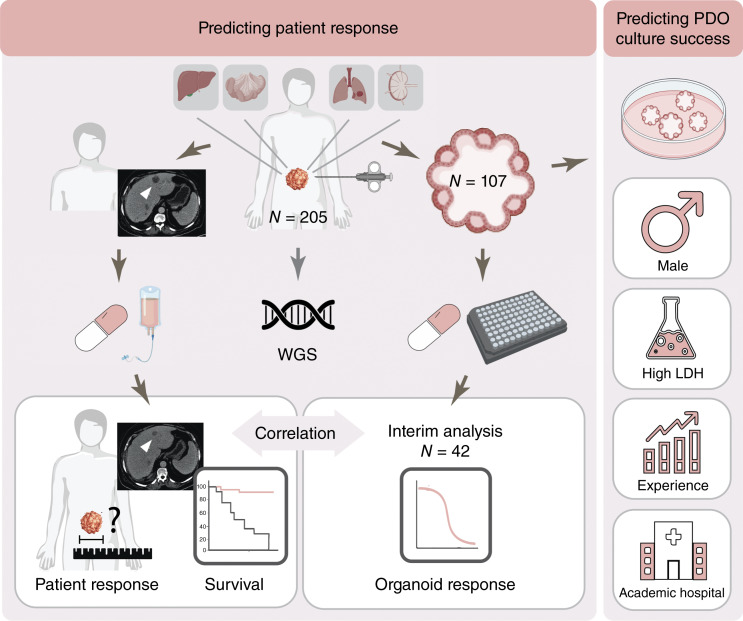
Schematic overview of the study. Patients underwent a biopsy of a metastasis for PDO culture and WGS, before starting a new line of systemic treatment. Predictors of successful PDO establishment were identified: male sex, increased LDH, biopsy in academic hospitals, optimized culture conditions, and experience. A total of 42 PDOs were screened for standard-of-care treatments, and the patient received standard systemic treatment. PDO response was correlated with patient response, and the association between PDO response and survival was assessed. (Created in BioRender. Roodhart, J. [2025] https://BioRender.com/by1f1uq.)

OPTIC is a Dutch multicenter observational cohort study (NL61668.041.17). This early-phase biomarker study evaluates the potential value of incorporating organoid technology in the stratification of patients with mCRC for treatment with antineoplastic agents. Outcome evaluation for* in vivo* response to treatment was performed blinded from *in vitro* data. The University Medical Center Utrecht collected the clinical data from the participating clinical centers in Castor Electronic Data Capture. HUB Organoids B.V. generated the PDO response data from coded patients. Patients underwent a biopsy procedure of a metastatic lesion and a blood withdrawal before the start of treatment. The study included patients with mCRC who received standard-of-care treatment with chemotherapy and/or targeted agents, regardless of the line of treatment. Evaluable treatments included but were not limited to capecitabine, 5-FU, or S-1 combined with oxaliplatin or irinotecan and/or anti-EGFR therapy [CAPOX, FOLFOX(-P), FOLFIRI(-P), and S-1 and oxaliplatin], capecitabine monotherapy, 5-FU monotherapy, irinotecan monotherapy, anti-EGFR monotherapy (panitumumab and cetuximab), and trifluridine/tipiracil. Combinations with bevacizumab were allowed. Eligibility criteria included consent for the Prospective Dutch Colorectal Cancer Cohort (PLCRC), >18 years of age, metastases localized outside the bone from which a biopsy can safely be obtained, measurable metastases on CT imaging, and no contraindications for lidocaine. Exclusion criteria included additional unrelated tumors influencing treatment decision-making, potentially affecting size changes of metastases, or competing risk for survival. The primary clinical endpoint was the response of the index metastasis to standard-of-care treatment that is biopsied for PDO culture, as measured by change in size on a CT scan. Secondary clinical endpoints were response according to RECIST 1.1, response of all metastatic target lesions, and dichotomized (decrease or increase) response. Lesion size was extracted from diagnostic reports and reassessed by a radiologist or oncologist if the biopsied lesion was not measured or measurements were not reported according to RECIST 1.1. The primary readout of the PDO test was a continuous measure. Based on prior PDO studies, a Pearson correlation coefficient of 0.35 was assumed for sample size calculation, targeting a power of 90% (*α* = 0.05) to detect an area under the ROC curve (AUROC) of 0.70, requiring 85 evaluable patients. Accounting for a 56% dropout rate due to failure of PDO culture or treatment initiation, 193 patients with biopsies sent for PDO culture should be enrolled. As some PDOs did not grow sufficiently for screening or patients were not clinically evaluable, a total of 205 patients with biopsies sent for PDO culture were enrolled. In this interim analysis, the first 42 PDOs are screened. PDOs were selected based on the availability of complete clinical data from the corresponding patients and the evaluability of treatment with 5-FU and oxaliplatin. For three PDOs, two treatments could be evaluated. PDOs established from <5 cells and those with only one PDO per evaluable treatment were not included in correlation analyses. Secondary objectives were evaluating the relationship between the PDO test and progression-free survival (PFS), evaluating the ability of the PDO test to discriminate between responders and nonresponders on the index metastasis level and patient level, and evaluating determinants of (un)successful PDO culture. A schematic overview of the study design is presented in Fig. 1. The medical ethical committee of the University Medical Center Utrecht approved the OPTIC trial. All patients participating in this study signed informed consent. The study was performed in accordance with the Declaration of Helsinki.

### Patient material processing and organoid culture

One or two 18-gauge biopsies from the same metastatic lesion were used to culture PDOs, which were subsequently incubated with the same standard-of-care treatment as has been given to the patient. One additional biopsy was used for whole genome sequencing (WGS) immediately or was stored at −80°C to enable DNA sequencing later. Biopsies were collected in advanced DMEM with nutrient mixture Ham F-12 (Ad-DF; #12634, Invitrogen), supplemented with 1% penicillin–streptomycin (#15140-122, Invitrogen), 1% HEPES [4-(2-hydroxyethyl)-1-piperazineethanesulfonic acid, #15630-056, Invitrogen], and 1% GlutaMAX (#35050, Invitrogen; hereafter referred to as Ad-DF+++), supplemented with 10 µmol/L Y-27632 (#M1817, AbMole).

PDO isolation was performed as described by van de Wetering and colleagues (7). PDOs were passaged using mechanical dissociation by pipetting and enzymatic dissociation with TrypLE Express (#12604021, Gibco) for 5 to 10 minutes at 37°C, washed using organoid culture medium, and re-plated by embedding in a solution of ice-cold Matrigel (#356231, Corning; 70%) and organoid culture medium and plated as droplets on a prewarmed six-well plate. After solidification of the Matrigel, organoid culture medium, containing Y-27632, was added to the plates. The organoid culture medium was refreshed three times per week. PDOs were expanded and cultured using media listed in Supplementary Table S1A and S1B. Confirmation of PDO identity was performed as described previously (16). After initial expansion, PDOs were cryopreserved until the end of the study to enable efficient and synchronized screening of multiple PDO lines within a short timeframe. The timeframe up to cryopreservation, plus 2 weeks of expansion for drug screening, reflects the typical duration to reach screening readiness, rather than the exact timepoint at which screening was performed. The total number of passages between collection and drug screens ranged from 7 to 25, with a median of 15. *Mycoplasma* testing was performed before the drug screen. Organoid culture medium without primocin and without refreshment for 5 to 7 days was taken from the culture and tested with MycoAlert Detection Kit (#LT07-318, Lonza). Research Resource Identifiers (RRIDs) of all PDOs are listed in Supplementary Table S2.

### Prediction model

To evaluate the impact of various factors on successful PDO establishment, we aimed to identify predictors. We first conducted univariate analyses, followed by multivariate analyses to determine if these predictors maintained independent predictive value. The following variables were evaluated as potential predictors: age, sex, location (rectum/left-sided/right-sided) and differentiation (poor/good/unknown) of the primary tumor, time to metastases (synchronous/metachronous), lactate dehydrogenase (LDH) level (below/above 250 U/L, based on the cutoff used in clinical practice), prior treatment (adjuvant/palliative/none), mutational status [(*KRAS/BRAF*/wild-type (WT)], date of biopsy (measured in days from first biopsy of the study), and hospital (academic/nonacademic). Time from first biopsy was categorized using knots determined based on data distribution and interventions (culture optimization, increase, and stabilization of inclusion rates). Only patients with complete data for all predictor variables were included in the analysis. To determine the optimal model, we applied backward stepwise selection using the Akaike information criterion to balance model fit and complexity. Multicollinearity was assessed using variance inflation factors, considering a variance inflation factor >5 as indicative of significant multicollinearity. Model performance was evaluated using the AUROC.

### Drug screening

The timeline of the drug screen was as follows: On day −1, PDOs were sheared and re-plated. On day 0, PDOs were harvested by incubating with 1 mg/mL Dispase II (#17105041, Life Technologies Europe B.V.) for 30 minutes at 37°C, washed twice using organoid culturing medium with Y-27632, and filtered using 100- and 20-μm mesh filters to collect the PDOs which have passed through the 100-μm filter but not through the 20 μm filter, to remove debris and single cells. PDOs were resuspended in organoid screening medium with Y-27632 with 5% basement membrane extract (#3533-010-02, Bio-Techne) for a final 125,000 PDOs/20 mL concentration. Drug screening was performed as described previously (16). Using an automated Multidrop Combi Reagent Dispenser (RRID: SCR_019329), 40 μL of PDO suspension was dispensed in clear-bottomed, black-walled 384-well plates with ultralow-attachment coating (#4588, Corning). A 10-point logarithmic concentration range of the drugs (Supplementary Table S3), positive control (staurosporine), and negative controls (0.5% PBS, 0.2% DMSO, or a combination of 0.5% PBS and 0.2% DMSO, depending on the solvent used) were dispensed in technical triplicates using a Tecan Fluent liquid handler. The following standard-of-care colorectal cancer drugs were screened: 5-FU, oxaliplatin, SN-38 (active metabolite of irinotecan), 5-FU and oxaliplatin, 5-FU and SN-38, trifluridine/tipiracil, and panitumumab. Drug combination screens were based on our previous findings showing that the optimal use of a “fixed” or “ratio” approach differed between oxaliplatin- and irinotecan-based combination treatments (16). The screens did not include bevacizumab, as its main effect is on the tumor vasculature and cannot be evaluated in PDOs. The organoid screening medium was composed of the organoid culture medium (Supplementary Table S1A) supplemented with 5 µmol/L Y-27632 (#M1817, AbMole). For oxaliplatin-based screens, we have removed N-acetylcysteine. On day 5, readouts were obtained by quantifying cell viability using CyQUANT Direct proliferation assay (Invitrogen, C7026, 3× concentrated, 20 μL/well) with PerkinElmer Operetta CLS (RRID: SCR_018810). A baseline readout was measured on day 0 in a separate plate with PDOs without treatment to calculate growth rate inhibition (GR) values. Drug screens were performed in technical triplicates and biological duplicates. The drug screen quality was analyzed by examining the Z′-factor. Screens were excluded from the analysis if Z′-factor was below 0.3 or if technical errors had occurred (e.g., dispensing error). The deviation of different biological replicates was calculated by summing the difference in GR values for all drug concentrations per curve. If the sum exceeded the predefined threshold of 1.7, the respective biological replicates were excluded, and a third and/or fourth run was performed. Bright-field images of every well on each plate were acquired to enable visual inspection, particularly when screens failed to meet these quality criteria. Viability and GR metric values were calculated (17). GR values range from 1 to −1, with 0 to 1 for partial growth inhibition, 0 for complete cytostasis, and 0 to −1 for cell death. The area under the nonfitted “curve” of the raw viability or GR values (AUC and GR_AUC_), and concentration that gives half-maximal viability or GR inhibition (IC_50_ and GR_50_) were calculated. Normalized values for GR and viability curve parameters were calculated using the maximum and minimum measured parameters per drug to compare different treatment types with different concentration ranges.

### WGS

Preferably within 24 hours, snap-frozen tumor and blood samples were shipped by courier to Hartwig for WGS. Paired tumor–normal WGS was performed at Hartwig on Illumina NovaSeq 6000 platforms (Illumina, RRID: SCR_016387) with a sequencing depth of >90× for tumor DNA and >30× for blood control DNA according to standard procedures as described previously (11, 16). A tumor percentage of 20% was required for reporting. Sequencing data were analyzed using a fully open-source in-house bioinformatic pipeline optimized for cancer genomics diagnostics (code available through github.com/hartwigmedical). All data generation and data analysis procedures were performed under ISO17025:2015 and ISO/NEN27001 accreditation.

### Statistical analysis

Pearson correlation tests were utilized to examine linear associations between continuous variables and minimize the impact of outliers. A two-sided *t* test or Mann–Whitney U test was applied to compare two groups. A Kruskal–Wallis test was applied to compare three groups, followed by pairwise comparisons using the Dunn test with the Bonferroni correction for multiple testing. A Fisher exact test was used for categorical comparisons when any expected count was below 10; otherwise, a *χ*^2^ test was applied. PDOs were classified as sensitive if the normalized GR_AUC_ of the 5-FU and oxaliplatin drug screen was below 0.63. This cutoff was defined previously based on the response rate of approximately 60% for patients treated with FOLFOX in the first line (3, 4). Diagnostic performance of the PDO screens was assessed by calculating the positive predictive value (PPV) and negative predictive value (NPV) and computing the ROC curve and the AUC. To estimate 95% confidence intervals (CI) for PPV and NPV, the Wilson score interval method was used. A Firth-corrected Cox proportional hazards regression analysis was performed to assess associations between PDO response and survival after 5-FU and oxaliplatin treatment. A two-sided Wald test was used to evaluate the significance of the HR in the Cox regression model. Kaplan–Meier survival curves were used to plot PFS, and differences between groups were assessed using the log-rank test. Analysis was performed in R (version 4.4.3, RRID: SCR_000432).

## Results

### Organoid establishment success

**Figure 2.**
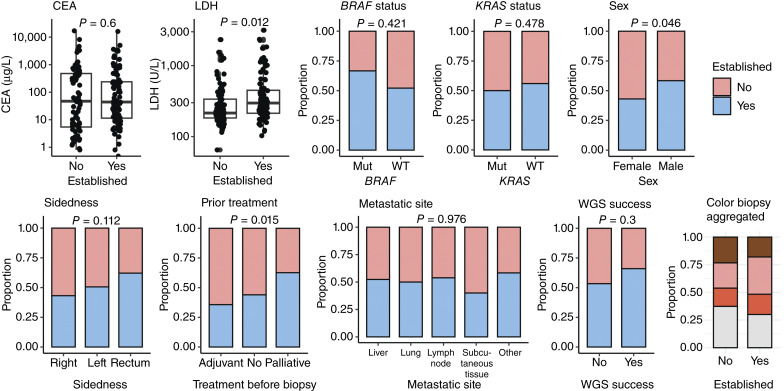
Clinical variables related to PDO establishment success: CEA, LDH, mutational status, sex, primary tumor sidedness, prior chemotherapeutic treatment, metastatic site of the biopsy, WGS success, and aggregated biopsy color (brown, pink, red, and white). CEA, carcinoembryonic antigen; mut, mutant.

There were 232 patient inclusions, with 9 patients being included multiple times. A total of 205 biopsies were taken. PDO establishment success was 52% overall (*n* = 107/205) and gradually increased from 22% in 2018 to 2020 to 75% in 2023 (Supplementary Fig. S1A and S1B). Critical factors in optimizing culture success were the replacement of several compounds in the culture medium (18), reducing time to processing and gaining more experience with handling biopsies. Specifically, success was enhanced by allowing more time to first split and by adopting an adaptive, observation-driven approach rather than adhering strictly to protocols. The different compounds in the original and adapted culture media are listed in Supplementary Tables S1A and S1B. Furthermore, using the first or second biopsy during the procedure for PDO culture was crucial for improving culture success, as its tissue quality was superior to that of later biopsies. All the abovementioned optimizations are captured in the period that the biopsy was taken. Univariable analyses show that patients with successfully established PDOs had significantly higher LDH levels than those with unsuccessful PDO cultures. Carcinoembryonic antigen levels, however, showed no association with PDO establishment. Additionally, success rates were higher for males and patients who received systemic palliative treatment prior to biopsy collection. The biopsies of successfully established PDOs contained a higher fraction of pink tissue (34% vs. 23%; Fig. 2). The distance from the hospital to the laboratory (range 0–142 km) had no impact on PDO establishment. Multivariable analyses on complete cases (175/204, one PDO received on dry ice was excluded) revealed that period of biopsy, sex, biopsy performed in an academic versus nonacademic hospital, and increased LDH were all independent predictors of PDO success (Supplementary Table S4). No significant multicollinearity or correlations were found between the predictive variables, indicating that each variable contributes unique information to the model. Prior systemic treatment was not a significant predictor in the final model. The model demonstrated good predictive performance, with an AUROC of 0.75. The minimum and maximum chances were 8% and 87% and the 25th and 75th percentiles were 34% and 70%, respectively. Calibration of the prediction model was assessed, showing good agreement between predicted and observed probabilities (Supplementary Fig. S2).

### Organoid and patient characteristics

Of the 107 PDOs, the first 42 were screened for different systemic treatments (Supplementary Tables S2 and S3) in this interim analysis. The median time from biopsy to sufficient expansion for biobanking was 61 days (range, 22–166), with an additional expansion of 2 weeks required for drug screening. Patients were selected based on the availability of complete clinical data and treatment with combination treatment with a fluoropyrimidine (5-FU, capecitabine, or S-1) and oxaliplatin (further referred to as 5-FU and oxaliplatin). The clinical characteristics of the full study population with established PDOs (*n* = 107) are shown in Supplementary Table S5. Representativeness of study participants is shown in Supplementary Table S6. The clinical characteristics of the population with screened PDOs in this interim analysis (*n* = 42) are shown in Table 1. More than half of the patients had received prior palliative chemotherapy when the PDO was established (22/42). The cohort includes 19 *KRAS*-mutant, five *BRAF*-mutant (of which one also had a subclonal *KRAS* mutation), and 18 *KRAS/BRAF*-WT tumors. The cohort comprises 19 patients with rectal tumors, 13 patients with left-sided tumors, and 10 patients with right-sided tumors. Among patients who received prior palliative chemotherapy (*n* = 22), all patients received fluoropyrimidines, 82% received oxaliplatin, 64% received irinotecan, and most patients received bevacizumab. The individual treatment courses, responses, progression, and biopsy dates are shown in Supplementary Fig. S3.

**Table 1.** Baseline characteristics of the study population, with key demographic, clinical, and molecular details from regular diagnostic sequencing.

Characteristic	Overall (*N* = 42)
Age	​
Median (min, max)	64 (37, 83)
Sex	​
Female	12 (28.6%)
Male	30 (71.4%)
Time to metastases	​
Metachronous	10 (23.8%)
Synchronous	32 (76.2%)
Site of biopsy	​
Liver	34 (81.0%)
Lung	1 (2.4%)
Lymph node	3 (7.1%)
Peritoneum/omentum	1 (2.4%)
Other	3 (7.1%)
Primary tumor location	​
Rectum (rectosigmoid/rectal)	19 (45.2%)
Left-sided (splenic flexure–sigmoid)	13 (31.0%)
Right-sided (cecum–transverse colon)	10 (23.8%)
Mutational status	​
*BRAF* mutation	5 (11.9%)
*KRAS* mutation	20 (47.6%)
WT	17 (40.5%)
MMR status	​
dMMR	1 (2.4%)
pMMR	41 (97.6%)
Prior systemic treatment	​
Adjuvant only	4 (9.5%)
None	16 (38.1%)
Palliative	22 (52.4%)
Type of prior palliative treatment	​
Fluoropyrimidine	22 (52.4%)
Oxaliplatin	18 (42.9%)
Irinotecan	14 (33.3%)

Diagnosis of metastatic disease after 6 months is considered metachronous. The other category includes subcutaneous and adnexal metastases.

Abbreviations: dMMR, deficient mismatch repair; max, maximum; min, minimum; MMR, mismatch repair; *N*, count; pMMR, proficient mismatch repair.

**Figure 3.**
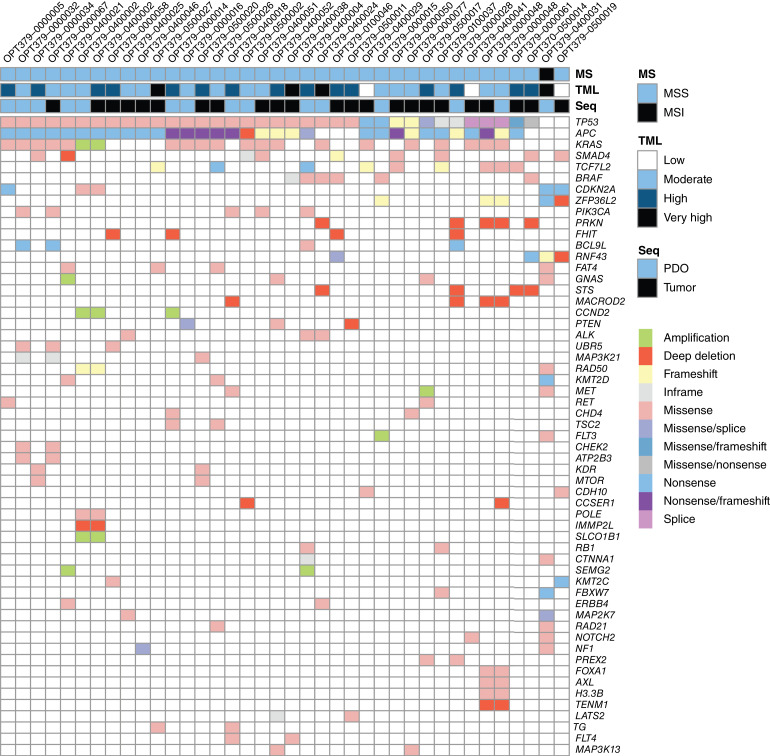
Genomic landscape of 36 tumors and/or PDOs derived from patients with mCRC. Top, microsatellite stability, tumor mutational load, tumor mutational burden, and whether the sequencing was performed on the PDOs or the tumor of origin; bottom, somatic driver mutations. MS, microsatellite status; MSI, microsatellite instability; MSS, microsatellite stability; Seq, sequencing; TML, tumor mutational load.

For 36 of 42 samples, the tumor or PDO was characterized by WGS. An overview of the driver genes with an aberration frequency of more than 5% is shown in Fig. 3. The PDO and matched tumor tissue were both sequenced for two patients (OPT379-0000048 and OPT379-0400002), and the driver landscape showed an overlap of 81% and 100%, respectively. *KRAS* and *BRAF* mutations were determined by regular diagnostics for all patients, with half determined on metastatic tissue and the other half on primary tumor tissue, not necessarily from the same lesion as the PDO biopsy. The concordance of the *KRAS* and *BRAF* mutational status from WGS and regular diagnostic sequencing was 94% (1 *KRAS* mutation and 1 *BRAF* mutation in WGS and WT in regular diagnostics, 2 *KRAS* mutations in regular diagnostics and WT in WGS, and 32 samples full concordance). As WGS was determined on the same metastatic tissue as the PDO, WGS results were leading in further analyses.

The quality of the PDO drug screening assay was evaluated using several measures. The mean Z′-factor for all PDOs and treatments was 0.63, indicating a good quality drug screen. To assess reproducibility, we compared drug screens of the same PDO culture performed in 2022 with 2024. The drug–response curves showed good overlap, confirming the reproducibility and robustness of the screens (Supplementary Fig. S4). All drug–response curves are shown in Supplementary Fig. S5.

### Organoids to predict treatment response

**Figure 4.**
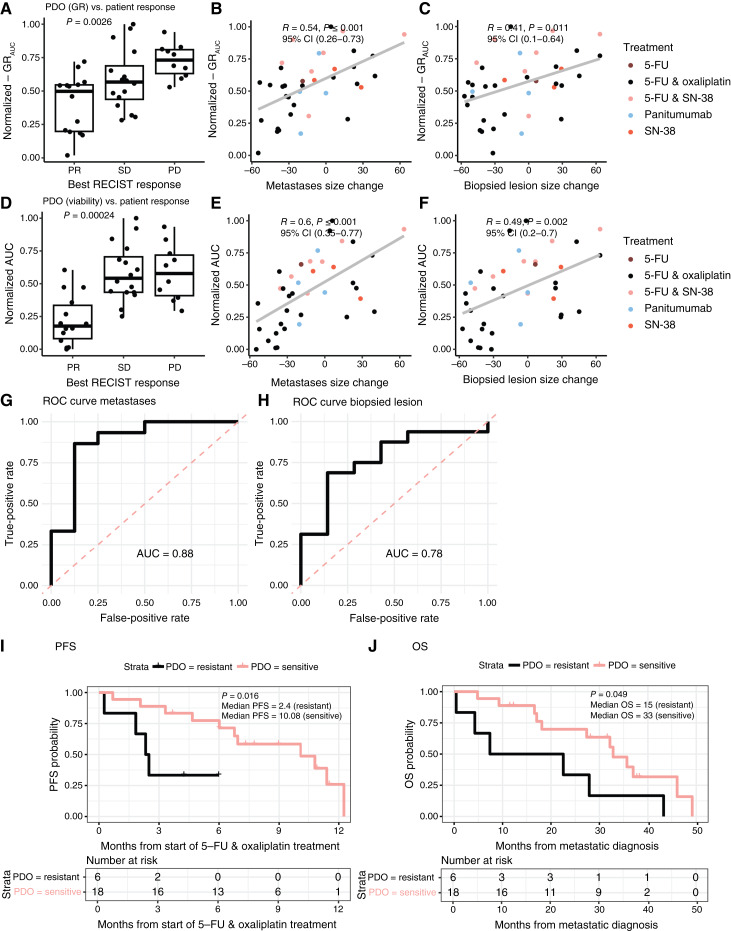
Correlations between patient response (best RECIST response, size change of all target lesions, and the biopsied lesion) and PDO response (normalized GR_AUC_ per treatment in **A–C** and AUC in **D–F**). ROC curves showing the performance of the PDO classification based on GR_AUC_. The curve plots the true-positive rate (sensitivity) against the false-positive rate (1 − specificity). The AUC indicates the model’s ability to distinguish between responders and nonresponders to 5-FU and oxaliplatin, defined by size change of all target lesions (**G**) and the biopsied lesion (**H**). Kaplan–Meier PFS (**I**) and OS (**J**) curves of patients stratified by PDO sensitivity to 5-FU and oxaliplatin, based on normalized GR_AUC_ (cutoff = 0.63). Censored events are indicated by vertical bars on the corresponding curve. The table underneath each plot denotes the numbers at risk. Log-rank test–based *P* value is shown. SN-38, active metabolite of irinotecan.

First, the primary and key secondary outcomes were evaluated for all treatments combined. PDO and patient response were compared specifically for the therapy regimen that the patient actually received. PDOs from patients with stable disease (SD) or progressive disease (PD), according to RECIST 1.1, were significantly more resistant *in vitro* compared with PDOs from patients with partial response (PR; GR_AUC_, 0.49 for PR and 0.57 for SD, 0.73 for PD; *P* = 0.003; Fig. 4A and D). Interestingly, PDO response (GR_AUC_) and response to systemic treatment on the patient level (sum of all target lesions) showed a better correlation (Pearson correlation, 0.54–0.60; *P* < 0.001; Fig. 4B and E) than the primary endpoint: response on the metastases level (biopsied lesion, Pearson correlation, 0.41–0.49; *P* < 0.01; Fig. 4C and F). The individual patient responses are shown in Supplementary Fig. S6. Next, a subgroup analysis was performed for the first-line treatment with 5-FU and oxaliplatin (*n* = 23). PDO response to 5-FU and oxaliplatin significantly reflected the response of all target lesions per best RECIST response category (GR_AUC_ 0.50 for PR, 0.45 for SD, and 0.73 for progressive disease; *P* = 0.006) and as a continuous measure (Pearson correlation, 0.58; *P* = 0.004; Supplementary Fig. S7). Drug–response curve parameters GR_50_ and IC_50_ are also commonly used to quantify drug sensitivity (19–21). These parameters were less predictive of patient outcomes (Supplementary Fig. S8) compared with GR_AUC_ and AUC.

For 23 patients treated with 5-FU and oxaliplatin, tumors were classified as either sensitive or resistant based on changes in size of all target lesions or the biopsied lesion (increase vs. decrease). PDOs were classified as sensitive or resistant based on the normalized GR_AUC_ of the 5-FU and oxaliplatin drug screen. The GR_AUC_ cutoff of 0.63, previously defined and tested based on a ∼60% response rate to first-line FOLFOX treatment (16), was reevaluated to validate its ability to predict response to 5-FU and oxaliplatin treatment. The 5-FU and oxaliplatin PDO screen demonstrated a PPV of 0.78 (14/18, 95% CI, 0.55–0.91) for predicting treatment sensitivity and an NPV of 0.80 (4/5, 95% CI, 0.38–0.96) for predicting treatment resistance based on all target lesions. For the biopsied lesion, the PPV remained 0.78 (14/18, 95% CI, 0.55–0.91), whereas the NPV was 0.60 (3/5, 95% CI, 0.23–0.88). The AUROC was 0.88 (95% CI, 0.71–1.00) for all target lesions and 0.78 (95% CI, 0.56–0.99) for the biopsied lesion (Fig. 4G and H).

### Organoids to predict (progression-free) survival after treatment with 5-FU and oxaliplatin

We assessed PFS as a secondary outcome for patient response to the most common treatment in the first line: 5-FU and oxaliplatin. We found that PDO response to 5-FU and oxaliplatin as a continuous measure (GR_AUC_) was significantly associated with PFS after the start of 5-FU and oxaliplatin treatment in Firth-corrected Cox regression analysis (HR, 1.35 for each 0.1 GR_AUC_ increase; 95% CI, 1.05–1.75; *P* = 0.021), under the assumption of linearity. Second, we used the predefined PDO classification as sensitive or resistant based on the GR_AUC_. PFS of patients with PDOs classified as sensitive was substantially longer compared with that of patients with resistant PDOs (median PFS, 10.1 vs. 2.4 months; *P* = 0.016; Fig. 4I). Similarly, we assessed if PDOs could predict OS. We found that PDO response to 5-FU and oxaliplatin as a continuous measure (GR_AUC_) was significantly associated with OS (from metastatic diagnosis) after 5-FU and oxaliplatin treatment in univariable Cox regression sensitivity analysis (HR, 1.27 for each 0.1 GR_AUC_ increase; 95% CI, 1.01–1.64; *P* = 0.049). OS of patients with PDOs classified as sensitive was substantially longer compared with that of patients with resistant PDOs (median OS, 33 vs. 15 months; log-rank *P* = 0.049; Fig. 4J).

**Figure 5.**
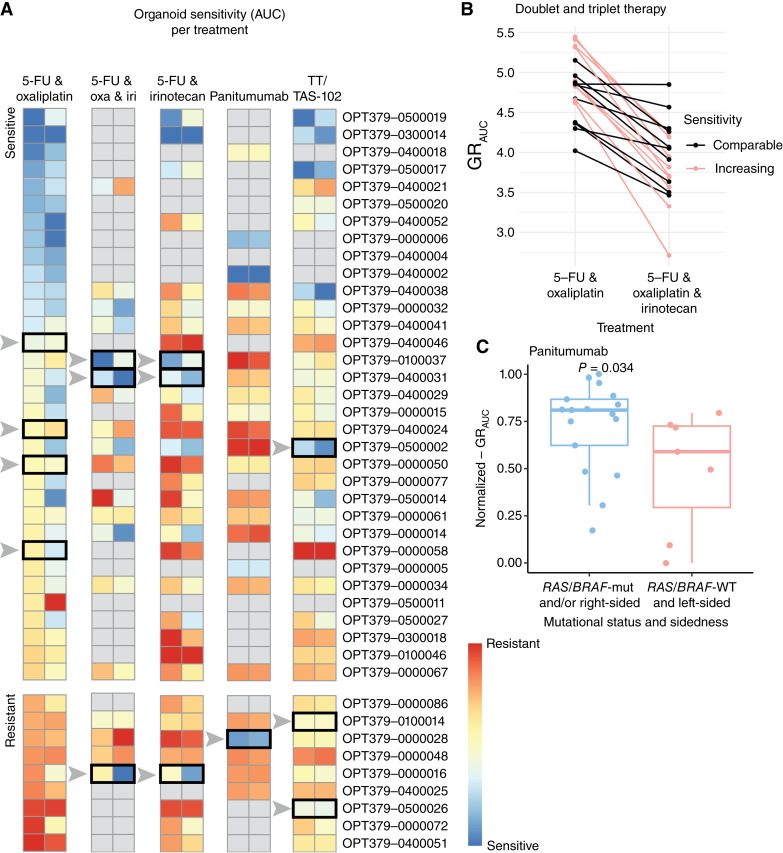
**A,** Heatmap of the drug sensitivity per PDO for different types of systemic treatment. The columns show the normalized GR_AUC_ (left) and AUC (right), respectively. Potential other treatment options for intermediate or high 5-FU and oxaliplatin–resistant PDOs, as well as intermediate or high 5-FU and oxaliplatin–sensitive but 5-FU and irinotecan–resistant PDOs, are shown in black bars. PDOs that were not screened are shown in gray. **B,** Sensitivity (GR_AUC_) to doublet and triplet chemotherapy showing increased sensitivity with the addition of irinotecan. **C,** Sensitivity (normalized GR_AUC_) to panitumumab for PDOs categorized according to the tumor’s mutational status and primary tumor sidedness (*RAS*-mutant, *BRAF*-mutant, and/or right-sided; *RAS/BRAF*-WT and left-sided. Boxplots show the minimum, median, maximum, upper and lower quartiles, and individual data points. iri, irinotecan; oxa, oxaliplatin; TT, trifluridine/tipiracil.

For patients with resistant PDOs to 5-FU and oxaliplatin, other potential treatment options could be identified by the PDO drug screens (Fig. 5A). Although we observed co-sensitivity for different types of systemic treatments, we identified some PDOs with resistance to 5-FU and oxaliplatin that showed sensitivity to other standard-of-care treatments like 5-FU and irinotecan, panitumumab, or trifluridine/tipiracil. Four patients with resistant PDOs would potentially benefit from other treatments, and two PDOs with intermediate sensitivity showed higher sensitivity to 5-FU and irinotecan or triplet chemotherapy, and another to trifluridine/tipiracil. It should be noted that PDO sensitivity is normalized per type of treatment, and the response rate of trifluridine/tipiracil in clinical settings is typically lower than that of 5-FU and oxaliplatin or 5-FU and irinotecan. The increase in sensitivity to triplet therapy was assessed using raw GR_AUC_ values rather than normalized values, showing a clear benefit in about half of the PDOs (Fig. 5B).

### Validation of standard-of-care patient selection for EGFR inhibition

In standard clinical practice, patients are selected for EGFR inhibitors based on *RAS/BRAF* mutation status and location of the primary tumor. We investigated the impact of genomic biomarkers and sidedness on PDO drug sensitivity. We found that PDOs obtained from patients with a left-sided *RAS/BRAF*-WT tumor (*n* = 9) were more sensitive to EGFR inhibitor panitumumab, compared with PDOs obtained from patients with right-sided and/or *RAS/BRAF*-mutant tumors (*n* = 15; median-normalized GR_AUC_, 0.59 vs. 0.81; *P* = 0.012; Fig. 5C).

## Discussion

In this interim analysis of a prospective clinical study, we validated that mCRC PDOs can predict patient outcomes during systemic treatment, specifically with the most common treatment 5-FU and oxaliplatin. Optimizing medium components, reducing biopsy processing time, and increasing experience were key to improving PDO culture success from needle biopsies. This optimization was captured in the time between the start of the study and the biopsy. However, other factors also influence culture success. Besides the period that the biopsy was taken, male sex, increased LDH level, and biopsy in an academic hospital were all independently predictive for high PDO establishment rates. An increased LDH level illustrates that aggressive tumor characteristics may favor PDO culture success. The unexpected finding that male sex has additional predictive value for higher PDO establishment rates might be due to the absence of estrogen in the culture medium, which might negatively affect the growth of female PDOs and warrants further investigation. PDO culture success was higher for patients who received systemic treatment before the biopsy. Prior systemic treatment was, however, not an independent predictor for PDO culture success in the multivariable model. This may be due to changes in inclusion criteria for later-line treatments, which were introduced along with optimization efforts, potentially related to the period when the biopsy was taken. Although the multivariable model showed good discrimination between successful and unsuccessful PDO cultures, there is a potential for overfitting. The results may be specific to the current dataset, requiring external validation. Prior studies found that biopsies from liver metastases showed the highest success rates, whereas deficient mismatch repair, *BRAF*-mutant tumors and prior chemotherapy exposure were associated with low success rates, contrasting with our findings (13–15, 19). Tumor biopsies are known to be more challenging to culture than resection specimens because of limited amount of available material (14). Nevertheless, up to 80% success rates can be achieved in growing PDOs from tumor biopsies (19, 22). Shifting toward biopsy-derived PDO establishment would be clinically relevant, making this approach accessible to more patients and accelerating clinical implementation.

We validated the predictive value of PDOs for stratifying patients receiving chemotherapy, specifically using a predefined cutoff for drug sensitivity. Applying this threshold in the PDO screen yielded a high NPV and PPV. The NPV confirms that resistant tumors are accurately identified, helping avoid unnecessary exposure to ineffective treatments and associated toxicities. Potential reasons for false positives may include resistance mechanisms within the tumor microenvironment or adaptive responses that are not fully captured in PDO models. In other studies, AUROC for predicting response to 5-FU and oxaliplatin ranged from 0.80 to 0.89, and PPV and NPV ranged from 88% to 90% and 74% to 100%, respectively (11, 12). Prospective studies also support these results, although this involves only small cohorts with clinical data (22). The improved predictive value compared with other studies could be due to our application of several drug screen optimization steps, including the removal of N-acetylcysteine from the screening medium, which interferes with oxaliplatin (16, 23, 24). Moreover, studies that used comparable 5-FU and oxaliplatin ratios with those used in our screens also reported good correlations with patient response (19, 25, 26). Our data showed that PDOs predicted treatment response for all target metastases and not just the one biopsied. This suggests that PDOs can capture tumor heterogeneity, making them a valuable tool for clinical application, as they may provide a broader, more accurate representation of treatment response across multiple metastatic sites. We observed a significant and clinically relevant difference in PFS and OS of patients treated with 5-FU and oxaliplatin, based on PDO predictions. This difference in survival highlights the utility of the validated cutoff in predicting treatment efficacy. Together, these results provide a reliable basis for interpreting 5-FU and oxaliplatin sensitivity in PDO screens, supporting their use in personalized cancer therapies.

The transition from doublet chemotherapy regimens to the triplet regimen FOLFOXIRI has altered the role of chemotherapy sensitivity testing. It could be applied for patients unfit for triplet therapy, for later treatment lines, or as part of a de-escalation strategy. When an *in vitro* response to one or more components is lacking, it can aid in sparing patients the unnecessary toxicity. For patients with PDOs resistant to 5-FU and oxaliplatin, drug screens could identify alternative treatment options, highlighting sensitivity to other chemotherapeutic or targeted therapies. Despite drug co-resistance in most cases, our data show that seven PDOs exhibited selective sensitivity to alternative treatments, suggesting potential benefit from different regimens. Four PDOs showed selective sensitivity to 5-FU and oxaliplatin. This aligns with prior studies reporting that a subset of resistant PDOs retain sensitivity to at least one drug, highlighting the clinical relevance of individualized drug profiling (27, 28). However, to date, prospective PDO-guided intervention trials have only been performed on a limited scale. The increased sensitivity of PDOs derived from left-sided *RAS/BRAF*-WT tumors to panitumumab confirms our previous observations (16), reinforcing that tumor characteristics influencing targeted drug response are preserved in PDOs. Research into additional predictive biomarkers for EGFR inhibition, beyond mutation status and tumor sidedness, is ongoing (29).

A limitation of our study is that we did not have large cohorts of evaluable patients for all treatments, which may impact the generalizability of the findings for specific treatments. However, by screening a substantial number of 42 PDOs, we could define a sensitivity window for each treatment, which could be analyzed across different treatments through normalization. The full cohort consists of 107 PDOs, with 85 evaluable PDOs to meet the predefined sample size. The remaining evaluable PDOs in the study will be included in future analyses. The moderate correlation coefficients observed between PDO response and metastatic size change indicate a significant, yet not particularly strong, association. Although this suggests that PDOs can capture most aspects of the tumor’s sensitivity, it also highlights the complexity of translating *in vitro* findings to *in vivo* outcomes. Factors like patient-specific drug metabolism could contribute to this discrepancy. Potential selection biases during PDO establishment could also affect the predictive power of these models. Furthermore, the suitability of CT scan evaluation as the gold standard for comparing PDO response is debatable, as it primarily measures tumor size rather than viability. PET-CT and biomarkers like ctDNA could provide complementary insights into tumor viability and treatment response. One of the main challenges in integrating PDO drug screening for response prediction into clinical workflows is the time required for PDO expansion and subsequent drug screening. Typically, PDO culture from patient-derived tumors takes several weeks but can range from days up to a month. For patients with mCRC with urgent needs for therapeutic decisions, this timeframe presents a significant hurdle. Recent advancements in miniaturized high-throughput screening aim to accelerate the time from biopsy to actionable results, making the process more compatible with the rapid treatment needs of patients with mCRC (15).

Together, our findings validate PDOs as reliable tools for predicting treatment response with high accuracy, particularly to 5-FU and oxaliplatin. PDO sensitivity correlates with tumor shrinkage and survival, highlighting its potential in guiding personalized cancer therapy. Optimized culture conditions and experience have significantly improved PDO establishment success. To establish the clinical utility of PDO drug screens, a PDO-directed treatment trial is essential to show the impact on patient outcomes.

## Supplementary Material

Supplementary Data 1 Tables S1-S6 and Figures S1–S8

## Data Availability

WGS data are stored in the Hartwig databank. Data can be requested via https://www.hartwigmedicalfoundation.nl/data/aanvragen-data/. Sequencing data are not publicly available, as this would compromise patient consent. All other data generated in this study are available upon reasonable request to the corresponding author. The organoid models are available and can be requested via Foundation Hubrecht Organoid Biobank (https://www.hubrechtorganoidbiobank.org/).

## References

[1] Cervantes A , Adam R, Roselló S, Arnold D, Normanno N, Taïeb J, . Metastatic colorectal cancer: ESMO Clinical Practice Guideline for diagnosis, treatment and follow-up. Ann Oncol 2023;34:10–32. 36307056 10.1016/j.annonc.2022.10.003

[2] Morris VK , Kennedy EB, Baxter NN, Benson AB 3rd, Cercek A, Cho M, . Treatment of metastatic colorectal cancer: ASCO guideline. J Clin Oncol 2023;41:678–700. 36252154 10.1200/JCO.22.01690 PMC10506310

[3] Venook AP , Niedzwiecki D, Lenz HJ, Innocenti F, Fruth B, Meyerhardt JA, . Effect of first-line chemotherapy combined with cetuximab or bevacizumab on overall survival in patients with KRAS wild-type advanced or metastatic colorectal cancer: a randomized clinical trial. JAMA 2017;317:2392–401. 28632865 10.1001/jama.2017.7105 PMC5545896

[4] Yamazaki K , Nagase M, Tamagawa H, Ueda S, Tamura T, Murata K, . Randomized phase III study of bevacizumab plus FOLFIRI and bevacizumab plus mFOLFOX6 as first-line treatment for patients with metastatic colorectal cancer (WJOG4407G). Ann Oncol 2016;27:1539–46. 27177863 10.1093/annonc/mdw206

[5] Abraham JP , Magee D, Cremolini C, Antoniotti C, Halbert DD, Xiu J, . Clinical validation of a machine-learning-derived signature predictive of outcomes from first-line oxaliplatin-based chemotherapy in advanced colorectal cancer. Clin Cancer Res 2021;27:1174–83. 33293373 10.1158/1078-0432.CCR-20-3286

[6] Sato T , Vries RG, Snippert HJ, van de Wetering M, Barker N, Stange DE, . Single Lgr5 stem cells build crypt-villus structures in vitro without a mesenchymal niche. Nature 2009;459:262–5. 19329995 10.1038/nature07935

[7] van de Wetering M , Francies HE, Francis JM, Bounova G, Iorio F, Pronk A, . Prospective derivation of a living organoid biobank of colorectal cancer patients. Cell 2015;161:933–45. 25957691 10.1016/j.cell.2015.03.053 PMC6428276

[8] Sato T , Stange DE, Ferrante M, Vries RG, Van Es JH, Van den Brink S, . Long-term expansion of epithelial organoids from human colon, adenoma, adenocarcinoma, and Barrett’s epithelium. Gastroenterology 2011;141:1762–72. 21889923 10.1053/j.gastro.2011.07.050

[9] Huch M , Gehart H, van Boxtel R, Hamer K, Blokzijl F, Verstegen MM, . Long-term culture of genome-stable bipotent stem cells from adult human liver. Cell 2015;160:299–312. 25533785 10.1016/j.cell.2014.11.050 PMC4313365

[10] Clevers H . Modeling development and disease with organoids. Cell 2016;165:1586–97. 27315476 10.1016/j.cell.2016.05.082

[11] Sakshaug BC , Folkesson E, Haukaas TH, Visnes T, Flobak Å. Systematic review: predictive value of organoids in colorectal cancer. Sci Rep 2023;13:18124. 37872318 10.1038/s41598-023-45297-8 PMC10593775

[12] Wensink GE , Elias SG, Mullenders J, Koopman M, Boj SF, Kranenburg OW, . Patient-derived organoids as a predictive biomarker for treatment response in cancer patients. NPJ Precis Oncol 2021;5:30. 33846504 10.1038/s41698-021-00168-1 PMC8042051

[13] Li X , Larsson P, Ljuslinder I, Öhlund D, Myte R, Löfgren-Burström A, . Ex vivo organoid cultures reveal the importance of the tumor microenvironment for maintenance of colorectal cancer stem cells. Cancers (Basel) 2020;12:923. 32290033 10.3390/cancers12040923 PMC7226030

[14] Zeng YL , Wang SD, Li YR, Xue WS, Wang T, Tang YT, . Analysis of factors influencing the success rate of organoid culture in 1231 cases of colorectal cancer. Zhonghua Wei Chang Wai Ke Za Zhi 2023;26:780–6. 37574295 10.3760/cma.j.cn441530-20221128-00499

[15] Tan T , Mouradov D, Lee M, Gard G, Hirokawa Y, Li S, . Unified framework for patient-derived, tumor-organoid-based predictive testing of standard-of-care therapies in metastatic colorectal cancer. Cell Rep Med 2023;4:101335. 38118423 10.1016/j.xcrm.2023.101335 PMC10783557

[16] Smabers LP , Wensink E, Verissimo CS, Koedoot E, Pitsa KC, Huismans MA, . Organoids as a biomarker for personalized treatment in metastatic colorectal cancer: drug screen optimization and correlation with patient response. J Exp Clin Cancer Res 2024;43:61. 38414064 10.1186/s13046-024-02980-6 PMC10898042

[17] Hafner M , Niepel M, Chung M, Sorger PK. Growth rate inhibition metrics correct for confounders in measuring sensitivity to cancer drugs. Nat Methods 2016;13:521–7. 27135972 10.1038/nmeth.3853 PMC4887336

[18] Hanyu H , Sugimoto S, Sato T. Visualization of differentiated cells in 3D and 2D intestinal organoid cultures. Methods Mol Biol 2023;2650:141–53. 37310630 10.1007/978-1-0716-3076-1_12

[19] Wang T , Pan W, Zheng H, Zheng H, Wang Z, Li JJ, . Accuracy of using a patient-derived tumor organoid culture model to predict the response to chemotherapy regimens in stage IV colorectal cancer: a blinded study. Dis Colon Rectum 2021;64:833–50. 33709991 10.1097/DCR.0000000000001971

[20] Lee SH , Kim K, Lee E, Lee K, Ahn KH, Park H, . Prediction of TKI response in EGFR-mutant lung cancer patients-derived organoids using malignant pleural effusion. NPJ Precis Oncol 2024;8:111. 38773241 10.1038/s41698-024-00609-7 PMC11109121

[21] Le Compte M , Cardenas De La Hoz E, Peeters S, Smits E, Lardon F, Roeyen G, . Multiparametric tumor organoid drug screening using widefield live-cell imaging for bulk and single-organoid analysis. J Vis Exp 2022;(190):e64434. 10.3791/64434 36622028

[22] Cartry J , Bedja S, Boilève A, Mathieu JRR, Gontran E, Annereau M, . Implementing patient derived organoids in functional precision medicine for patients with advanced colorectal cancer. J Exp Clin Cancer Res 2023;42:281. 37880806 10.1186/s13046-023-02853-4 PMC10598932

[23] Ooft SN , Weeber F, Dijkstra KK, McLean CM, Kaing S, van Werkhoven E, . Patient-derived organoids can predict response to chemotherapy in metastatic colorectal cancer patients. Sci Transl Med 2019;11:eaay2574. 31597751 10.1126/scitranslmed.aay2574

[24] Narasimhan V , Wright JA, Churchill M, Wang T, Rosati R, Lannagan TRM, . Medium-throughput drug screening of patient-derived organoids from colorectal peritoneal metastases to Direct personalized therapy. Clin Cancer Res 2020;26:3662–70. 32376656 10.1158/1078-0432.CCR-20-0073 PMC8366292

[25] Ganesh K , Wu C, O’Rourke KP, Szeglin BC, Zheng Y, Sauvé CEG, . A rectal cancer organoid platform to study individual responses to chemoradiation. Nat Med 2019;25:1607–14. 31591597 10.1038/s41591-019-0584-2 PMC7385919

[26] Tang Y , Wang T, Hu Y, Ji H, Yan B, Hu X, . Cutoff value of IC_50_ for drug sensitivity in patient-derived tumor organoids in colorectal cancer. iScience 2023;26:107116. 37426352 10.1016/j.isci.2023.107116 PMC10329174

[27] Martini G , Belli V, Napolitano S, Ciaramella V, Ciardiello D, Belli A, . Establishment of patient-derived tumor organoids to functionally inform treatment decisions in metastatic colorectal cancer. ESMO Open 2023;8:101198. 37119788 10.1016/j.esmoop.2023.101198 PMC10265597

[28] Jensen LH , Rogatto SR, Lindebjerg J, Havelund B, Abildgaard C, do Canto LM, . Precision medicine applied to metastatic colorectal cancer using tumor-derived organoids and in-vitro sensitivity testing: a phase 2, single-center, open-label, and non-comparative study. J Exp Clin Cancer Res 2023;42:115. 37143108 10.1186/s13046-023-02683-4 PMC10161587

[29] Xu C , Mannucci A, Esposito F, Oliveres H, Alonso-Orduña V, Yubero A, . An exosome-based liquid biopsy predicts depth of response and survival outcomes to cetuximab and panitumumab in metastatic colorectal cancer: the EXONERATE Study. Clin Cancer Res 2025;31:1002–15. 39820673 10.1158/1078-0432.CCR-24-1934 PMC11913580

