# Supplementary material for: Patient-Derived Organoids Predict Treatment Response in Metastatic Colorectal Cancer
Source: Clin Cancer Res. 2025 Sep 22;31(23):5015–26. doi: 10.1158/1078-0432.CCR-25-1564 (PMC12666313; doi:10.1158/1078-0432.CCR-25-1564)
Supplement: Supplementary Data 1 — Tables S1-S6 and Figures S1–S8 [file ccr-25-1564_supplementary_data_1_suppds1.docx]

**Supplementary data**

| Component | Source (catalogue number) | Concentration |
| --- | --- | --- |
| Advanced (DMEM/F12) medium | Gibco (12634-010) | 1x |
| HEPES Buffer | Lonza (17737E) | 10 mM |
| Penicillin/Streptomycin | Gibco (15070-063) | 50 U/mL |
| GlutaMAX | Gibco (35050-038) | 2 mM |
| N-acetylcysteine (NAC) | Sigma-Aldrich (A9165) | 1.25 mM |
| A83-01 | SignalChem (A09-900-05) | 500 nM |
| B27 | Invitrogen (17504-044) | 1x |
| Human recombinant EGF | PeptroTech EC Ltd (A10187) | 50 ng/mL |
| Gastrin | Sigma-Aldrich (G9145) | 5 nM |
| Noggin-Fc conditioned medium | U-Protein Express BV (N002) | 1% vol/vol |
| Recombinant human R-spondin-3 conditioned medium | Bio-Techne Ltd (3500-RS/CFMTO) | 250 ng/mL |
| SB202190 | Gentaur (A1632) | 10 μM |
| Nicotinamide | Merck Life Science (N0636-100G) | 10 mM |
| Primocin | InvivoGene SAS (ANT-PM-2) | 50 μg/mL |
| Wnt surrogate | U-Protein Express BV (N001) | 0.25 nM |

| Component | Source (catalogue number) | Concentration |
| --- | --- | --- |
| Advanced (DMEM/F12) medium | Gibco (12634-010) | 50% |
| HEPES Buffer | Lonza (17737E) | 10 mM |
| Penicillin/Streptomycin | Gibco (15070-063) | 50 U/mL |
| GlutaMAX | Gibco (35050-038) | 2 mM |
| N-acetylcysteine (NAC) | Sigma-Aldrich (A9165) | 1.25 mM |
| A83-01 | SignalChem (A09-900-05) | 500 nM |
| B27 | Invitrogen (17504-044) | 1x |
| Human recombinant EGF | PeptroTech EC Ltd (A10187) | 50 ng/mL |
| Human recombinant IGF-I (carrier-free) | PeptroTech EC Ltd (100-11) | 100 ng/mL |
| Recombinant human FGF-2-basic | PeptroTech EC Ltd (100-18B) | 50 ng/mL |
| Noggin-Fc conditioned medium | U-Protein Express BV (N002) | 1% vol/vol |
| Recombinant human R-spondin-3 conditioned medium | Bio-Techne Ltd (3500-RS/CFMTO) | 250 ng/mL |
| Y-27632 (Rho-kinase) | Abmole (M1817) | 10 µM |
| Primocin | InvivoGene SAS (ANT-PM-2) | 50 μg/mL |
| Wnt conditioned medium |  | 50% |

**Supplementary Table S1. A)** Composition of original organoid culture medium. **B)** Composition of adapted organoid culture medium. Abbreviations: DMEM/F12 (Dulbecco’s Modified Eagle Medium/Ham’s F-12), FGF-2 (fibroblast growth factor), EGF (epidermal growth factor), IGF (insulin-like growth factor), mL (milliliter), mM (millimolar), nM (nanomolar), ng/mL (nanogram per milliliter), U/mL (Units per milliliter), μg/mL (microgram per milliliter), μM (micromolar), vol/vol (volume/volume).

| PDO | Treatment | Screened | Passage number | RRID |
| --- | --- | --- | --- | --- |
| OPT379-0000005 | 5-FU & oxaliplatin | Yes | 21 | CVCL_F0LH |
| OPT379-0000006 | 5-FU & oxaliplatin | Yes | 15 | CVCL_F0LI |
| OPT379-0000014 | 5-FU & oxaliplatin | Yes | 13 | CVCL_F0LJ |
| OPT379-0000016 | 5-FU & oxaliplatin | Yes | 12 | CVCL_F0LL |
| OPT379-0000028 | 5-FU & oxaliplatin | Yes | 15 | CVCL_F0LN |
| OPT379-0000048 | 5-FU & oxaliplatin | Yes | 15 | CVCL_F0LX |
| OPT379-0000050 | 5-FU & oxaliplatin | Yes | 12 | CVCL_F0LY |
| OPT379-0000061 | 5-FU & oxaliplatin | Yes | 12 | CVCL_F0M5 |
| OPT379-0000067 | 5-FU & oxaliplatin | Yes | 12 | CVCL_F0M7 |
| OPT379-0000077 | 5-FU & oxaliplatin | Yes | 15 | CVCL_F0ME |
| OPT379-0100046 | 5-FU & oxaliplatin | Yes | 13 | CVCL_F0MX |
| OPT379-0300014 | 5-FU & oxaliplatin | Yes | 19 | CVCL_F0N3 |
| OPT379-0400002 | 5-FU & oxaliplatin | Yes | 12 | CVCL_F0N8 |
| OPT379-0400004 | 5-FU & oxaliplatin | Yes | 20 | CVCL_F0N9 |
| OPT379-0400029 | 5-FU & oxaliplatin | Yes | 20 | CVCL_F0NL |
| OPT379-0400031 | 5-FU & oxaliplatin | Yes | 10 | CVCL_F0NN |
| OPT379-0400038 | 5-FU & oxaliplatin | Yes | 14 | CVCL_F0NT |
| OPT379-0400041 | 5-FU & oxaliplatin | Yes | 13 | CVCL_F0NW |
| OPT379-0400046 | 5-FU & oxaliplatin | Yes | 9 | CVCL_F0NZ |
| OPT379-0400051 | 5-FU & oxaliplatin | Yes | 17 | CVCL_F0P2 |
| OPT379-0400052 | 5-FU & oxaliplatin | Yes | 17 | CVCL_F0P3 |
| OPT379-0500002 | 5-FU & oxaliplatin | Yes | 10 | CVCL_F0P6 |
| OPT379-0500011 | 5-FU & oxaliplatin | Yes | 18 | CVCL_F0P8 |
| OPT379-0500014 | 5-FU & oxaliplatin | Yes | 11 | CVCL_F0P9 |
| OPT379-0500027 | 5-FU & oxaliplatin | Yes | 13 | CVCL_F0PI |
| OPT379-0100012 | 5-FU & oxaliplatin | No |  | CVCL_F0ML |
| OPT379-0000024 | 5-FU & oxaliplatin | No |  | CVCL_F0LM |
| OPT379-0100041 | 5-FU & oxaliplatin | No |  | CVCL_F0MV |
| OPT379-0000072 | 5-FU & oxaliplatin | Yes* | 21 | CVCL_F0MC |
| OPT379-0000015 | 5-FU & irinotecan | Yes | 18 | CVCL_F0LK |
| OPT379-0000034 | 5-FU & irinotecan | Yes | 13 | CVCL_F0LS |
| OPT379-0000058 | 5-FU & irinotecan | Yes | 25 | CVCL_F0M3 |
| OPT379-0000086 | 5-FU & irinotecan | Yes | 8 | CVCL_F0MK |
| OPT379-0100014 | 5-FU & irinotecan | Yes | 16 | CVCL_F0MM |
| OPT379-0500017 | 5-FU & irinotecan | Yes | 21 | CVCL_F0PA |
| OPT379-0500026 | 5-FU & irinotecan | Yes | 13 | CVCL_F0PH |
| OPT379-0100023 | 5-FU & irinotecan | No |  | CVCL_F0MQ |
| OPT379-0100037 | 5-FU & irinotecan | Yes** | 12 | CVCL_F0MT |
| OPT379-0300018 | 5-FU & irinotecan | Yes* | 18 | CVCL_F0N5 |
| OPT379-0400039 | 5-FU & irinotecan | No |  | CVCL_F0NU |
| OPT379-0400040 | 5-FU & irinotecan | No |  | CVCL_F0NV |
| OPT379-0400024 | Irinotecan | Yes | 15 | CVCL_F0NH |
| OPT379-0400031 | Irinotecan | Yes | 10 | CVCL_F0NN |
| OPT379-0500020 | Irinotecan | Yes | 15 | CVCL_F0PD |
| OPT379-0400056 | Irinotecan | No |  | CVCL_F0P5 |
| OPT379-0500019 | Irinotecan | Yes** | 18 | CVCL_F0PC |
| OPT379-0400021 | 5-FU/capecitabine | Yes | 15 | CVCL_F0NF |
| OPT379-0400008 | 5-FU/capecitabine | No |  | CVCL_F0NA |
| OPT379-0000039 | 5-FU/capecitabine | No |  | CVCL_F0LU |
| OPT379-0000006 | Panitumumab | Yes | 7 | CVCL_F0LI |
| OPT379-0000015 | Panitumumab | Yes | 16 | CVCL_F0LK |
| OPT379-0400018 | Panitumumab | Yes | 17 | CVCL_F0ND |
| OPT379-0400025 | Panitumumab | Yes | 13 | CVCL_F0NI |
| OPT379-0100019 | Panitumumab | No |  | CVCL_F0MN |
| OPT379-0300007 | Panitumumab | No |  | CVCL_F0MY |
| OPT379-0300010 | Panitumumab | No |  | CVCL_F0N1 |
| OPT379-0000051 | Panitumumab | No |  | CVCL_F0LZ |
| OPT379-0000057 | Panitumumab | No |  | CVCL_F0M2 |
| OPT379-0000079 | Panitumumab | No |  | CVCL_F0MF |
| OPT379-0000084 | Panitumumab | No |  | CVCL_F0MJ |
| OPT379-0300013 | TT | No |  | CVCL_F0N2 |
| OPT379-0300015 | TT | No |  | CVCL_F0N4 |
| OPT379-0400030 | TT | No |  | CVCL_F0NM |
| OPT379-0000053 | TT | No |  | CVCL_F0M0 |
| OPT379-0000069 | TT | No |  | CVCL_F0M9 |
| OPT379-0000073 | TT | No |  | CVCL_F0MD |
| OPT379-0400036 | TT | No |  | CVCL_F0NR |
| OPT379-0400042 | TT | No |  | CVCL_F0NX |
| OPT379-0500009 | TT | No |  | CVCL_F0P7 |
| OPT379-0500018 | TT | No |  | CVCL_F0PB |
| OPT379-0500021 | TT | No |  | CVCL_F0PE |
| OPT379-0500022 | TT | No |  | CVCL_F0PF |
| OPT379-0100021 | 5-FU & oxaliplatin & irinotecan | No |  | CVCL_F0MP |
| OPT379-0100026 | 5-FU & oxaliplatin & irinotecan | No |  | CVCL_F0MR |
| OPT379-0100038 | 5-FU & oxaliplatin & irinotecan | No |  | CVCL_F0MU |
| OPT379-0400013 | 5-FU & oxaliplatin & irinotecan | No |  | CVCL_F0NB |
| OPT379-0400019 | 5-FU & oxaliplatin & irinotecan | No |  | CVCL_F0NE |
| OPT379-0400023 | 5-FU & oxaliplatin & irinotecan | No |  | CVCL_F0NG |
| OPT379-0400026 | 5-FU & oxaliplatin & irinotecan | No |  | CVCL_F0NJ |
| OPT379-0000032 | 5-FU & oxaliplatin & irinotecan | Yes** | 14 | CVCL_F0LR |
| OPT379-0000042 | 5-FU & oxaliplatin & irinotecan | No |  | CVCL_F0LV |
| OPT379-0000065 | 5-FU & oxaliplatin & irinotecan | No |  | CVCL_F0M6 |
| OPT379-0400035 | 5-FU & oxaliplatin & irinotecan | No |  | CVCL_F0NQ |
| OPT379-0400037 | 5-FU & oxaliplatin & irinotecan | No |  | CVCL_F0NS |
| OPT379-0400048 | 5-FU & oxaliplatin & irinotecan | No |  | CVCL_F0P0 |
| OPT379-0500024 | 5-FU & oxaliplatin & irinotecan | No |  | CVCL_F0PG |
| OPT379-0300020 | 5-FU & irinotecan & panitumumab | No |  | CVCL_F0N7 |
| OPT379-0000031 | 5-FU & irinotecan & panitumumab | No |  | CVCL_F0LQ |
| OPT379-0000059 | 5-FU & irinotecan & panitumumab | No |  | CVCL_F0M4 |
| OPT379-0000081 | 5-FU & irinotecan & panitumumab | No |  | CVCL_F0MH |
| OPT379-0300019 | 5-FU & oxaliplatin & panitumumab | No |  | CVCL_F0N6 |
| OPT379-0000035 | 5-FU & oxaliplatin & panitumumab | No |  | CVCL_F0LT |
| OPT379-0000068 | 5-FU & oxaliplatin & panitumumab | No |  | CVCL_F0M8 |
| OPT379-0100032 | Sotorasib & panitumumab & chemotherapy | No |  | CVCL_F0MS |
| OPT379-0000071 | Sotorasib & panitumumab & chemotherapy | No |  | CVCL_F0MB |
| OPT379-0000044 | Encorafenib & cetuximab (& chemotherapy) | No |  | CVCL_F0LW |
| OPT379-0000055 | Encorafenib & cetuximab (& chemotherapy) | No |  | CVCL_F0M1 |
| OPT379-0000080 | Encorafenib & cetuximab (& chemotherapy) | No |  | CVCL_F0MG |
| OPT379-0000082 | Encorafenib & cetuximab (& chemotherapy) | No |  | CVCL_F0MI |
| OPT379-0300009 | Crizotinib | No |  | CVCL_F0N0 |
| OPT379-0400015 | Pembrolizumab | No |  | CVCL_F0NC |
| OPT379-0000070 | Trastuzumab & pertuzumab | No |  | CVCL_F0MA |
| OPT379-0100043 | None | No |  | CVCL_F0MW |
| OPT379-0300008 | None | No |  | CVCL_F0MZ |
| OPT379-0400028 | None | No |  | CVCL_F0NK |
| OPT379-0000030 | None | No |  | CVCL_F0LP |
| OPT379-0400034 | None | No |  | CVCL_F0NP |
| OPT379-0400043 | None | No |  | CVCL_F0NY |
| OPT379-0400050 | None | No |  | CVCL_F0P1 |
| OPT379-0400053 | None | No |  | CVCL_F0P4 |

**Supplementary Table S2.** The evaluable treatments after biopsy for all PDOs, categorized by whether they were screened or not. For screened PDOs, the passage number at the time of screening is indicated. Multiple treatments could be evaluated per PDO. *PDOs not included in correlation analyses, established from <5 cells. **PDOs not included in correlation analyses, evaluable treatment screen did not meet quality control, or was only available for one PDO. Abbreviations: 5-FU (5-fluorouracil), PDO (patient-derived organoid), TT (trifluridine/tipiracil).

| Drug | Source  (catalogue number) | Concentration range |
| --- | --- | --- |
| 5-FU | 15596885 (UMCU) | 0.05 – 900 µM |
| Oxaliplatin | 15532585 (UMCU) | 0.03 – 500 µM |
| SN-38 (irinotecan) | Selleck Chemicals GmbH (S4908) | 0.00001 – 1.8 µM |
| 5-FU & oxaliplatin ratio 1.8:1 | 15596885 (UMCU) + 15532585 (UMCU) | 5-FU: 0.05– 900 µM  Oxaliplatin: 0.03 – 500 µM |
| 5-FU & SN-38 (0.01) | 15596885 (UMCU) + Selleck Chemicals GmbH (S4908) | Anchor SN-38: 0.01 µM  5-FU: 0.01 – 2700 µM |
| TAS-102 | Selleck Chemicals GmbH (S8539) | 0.02 – 333 µM |
| Panitumumab (Vectibix) | 15343561 (UMCU) | 0.11 – 30000 ng/ml |
| Staurosporine | Merck Life Science N. V. (37095) | 2 µM |
| DMSO | VWR (ICNA0219605525) | 0.2% |
| PBS | Thermo Fisher Scientific (14190-144) | 0.5% |

**Supplementary Table S3.** Chemotherapies and targeted treatments used in drug screens. Abbreviations: 5-FU (5-fluorouracil), DMSO (dimethyl sulfoxide), PBS (phosphate buffered saline), SN-38 (active metabolite of irinotecan), μM (micromolar), UMCU (University Medical Center Utrecht).

|  | Univariable | | Multivariable | |
| --- | --- | --- | --- | --- |
| Predictor | **Odds Ratio (95% CI)** | **P-value** | **Odds Ratio (95% CI)** | **P-value** |
| Period biopsy (2) | 2.78 (1.01, 7.67) | 0.049 | 2.04 (0.69, 5.99) | 0.195 |
| Period biopsy (3) | 3.75 (1.44, 9.76) | 0.007 | 3.19 (1.17, 8.71) | 0.024 |
| Period biopsy (4) | 5.99 (2.35, 15.29) | <0.001 | 5.87 (2.17, 15.89) | <0.001 |
| Sex (Male) | 1.89 (1.02, 3.48) | 0.042 | 2.26 (1.14, 4.47) | 0.019 |
| LDH level (>250 U/L) | 2.45 (1.33, 4.51) | 0.004 | 2.12 (1.09, 4.12) | 0.026 |
| Hospital (Non-academic) | 0.44 (0.23, 0.85) | 0.015 | 0.36 (0.17, 0.76) | 0.007 |

**Supplementary Table S4.** Prediction model for PDO establishment success. Table showing the odds ratios (ORs) and 95% confidence intervals (CIs) from the final prediction model. The odds ratios represent the likelihood of the outcome in each category relative to the reference. Statistically significant results (p < 0.05) are indicated in bold. Period 1 started at the first inclusion in March 2018. Period 2 started with culture optimization in October 2020, period 3 started with an increase in inclusion rates in April 2022, and period 4 started when the inclusion rate stabilized in January 2023.

|  | Overall (N=107) |
| --- | --- |
| Age |  |
| Median (min, max) | 62 (37, 86) |
| Sex |  |
| Female | 34 (31.8%) |
| Male | 73 (68.2%) |
| Time to metastases |  |
| Metachronous | 29 (27.1%) |
| Synchronous | 78 (72.9%) |
| Site of biopsy |  |
| Liver | 89 (83.2%) |
| Lung | 2 (1.9%) |
| Lymph node | 7 (6.5%) |
| Peritoneum/omentum | 3 (2.8%) |
| Other | 6 (5.6%) |
| Primary tumor location |  |
| Rectum (rectosigmoid/rectal) | 41 (38.3%) |
| Left-sided (splenic flexure-sigmoid) | 44 (41.1%) |
| Right-sided (coecum-transverse colon) | 21 (19.6%) |
| Multiple primary tumors (with different sidedness) | 1 (0.9%) |
| Mutational status |  |
| *BRAF* mutation | 10 (9.3%) |
| *KRAS* mutation | 45 (42.1%) |
| Wildtype | 52 (48.6%) |
| MMR status |  |
| dMMR | 3 (2.8%) |
| pMMR | 103 (96.3%) |
| Unknown | 1 (0.9%) |
| Prior systemic treatment |  |
| Adjuvant only | 5 (4.7%) |
| None | 40 (37.4%) |
| Palliative | 62 (57.9%) |
| Type of prior palliative treatment |  |
| Fluoropyrimidine | 61 (57.0%) |
| Oxaliplatin | 52 (48.6%) |
| Irinotecan | 45 (42.1%) |

**Supplementary Table S5.** The baseline characteristics of the full study population, with key demographic, clinical, and molecular details from regular diagnostic sequencing. Diagnosis of metastatic disease after six months is considered metachronous. The other category includes abdominal, mediastinal, subcutaneous, and adnex metastases. Abbreviations: d/pMMR (deficient/ proficient mismatch repair), max (maximum), min (minimum), N (count), PDO (patient-derived organoid).

| Cancer type(s)/subtype(s)/stage(s)/condition | Metastatic colorectal cancer (mCRC) |
| --- | --- |
| Considerations related to: | |
| Sex | Overall mCRC incidence is higher in males. Younger females (18–44 years old) with mCRC live longer than younger males, older males (55 and older) live longer than older females. Right-sided tumors and *BRAF* mutations are more frequent among females. |
| Age | Median age at diagnosis is 69 years. 55% of CRC patients in the Netherlands are 55-75 years old. |
| Race/ethnicity | Hispanics and Asians with mCRC have better overall survival outcomes than Caucasians and African-Americans. |
| Geography | CRC is the third most common type of cancer in the Netherlands and worldwide. Incidence is higher in the Eastern regions and lower in the Southwestern regions compared to the average expected incidence in the Netherlands. CRC incidence and mortality are highest in Europe, Oceania, and North America. CRC incidence and mortality are rising in Eastern Europe, Asia, and South America, while stabilizing or declining in the USA and Western Europe, likely due to better early detection and prevention. |
| Other considerations | Colorectal cancer most often metastasizes to the liver. |
| Overall representativeness of this study | The age distribution of patients in this study is similar to the average age distribution of CRC in real-world data. The higher mCRC incidence in men in this study is representative of the higher mCRC incidence in real-world data. Caucasian ethnicity is overrepresented in this study. |

**Supplementary Table S6.** Representativeness of Study Participants


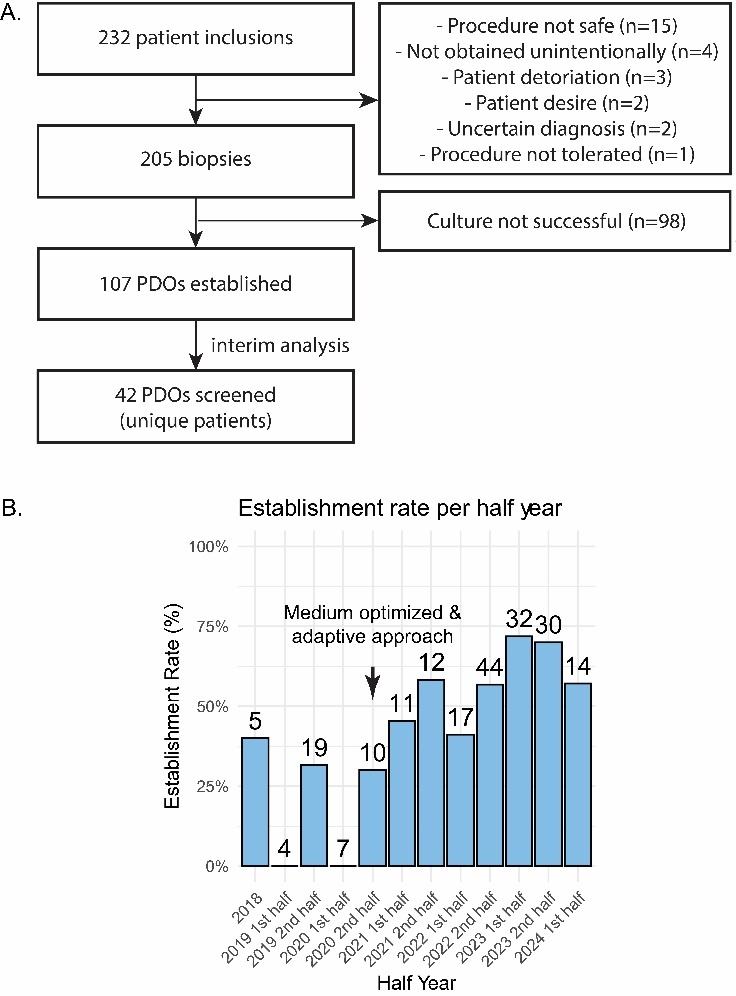


**Supplementary Figure S1. A)** Flowchart of the study. Of 232 patient inclusions, eight patients were included two times and one patient three times for different treatment lines. A total of 205 biopsies were taken from 196 unique patients. 107 PDOs were established, of which 42 were screened in this interim analysis. **B)** Establishment rate of patient-derived organoids from 2018-2024. The number of processed biopsies per half year is shown in the graph. Abbreviations: PDO (patient-derived organoid).


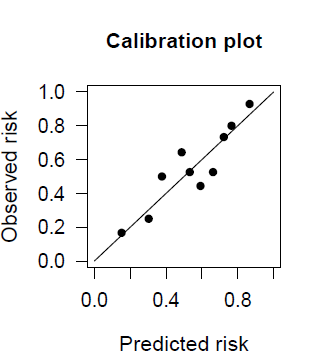


**Supplementary Figure S2.** Calibration of the prediction model for PDO establishment success shows good agreement between predicted and observed probabilities.


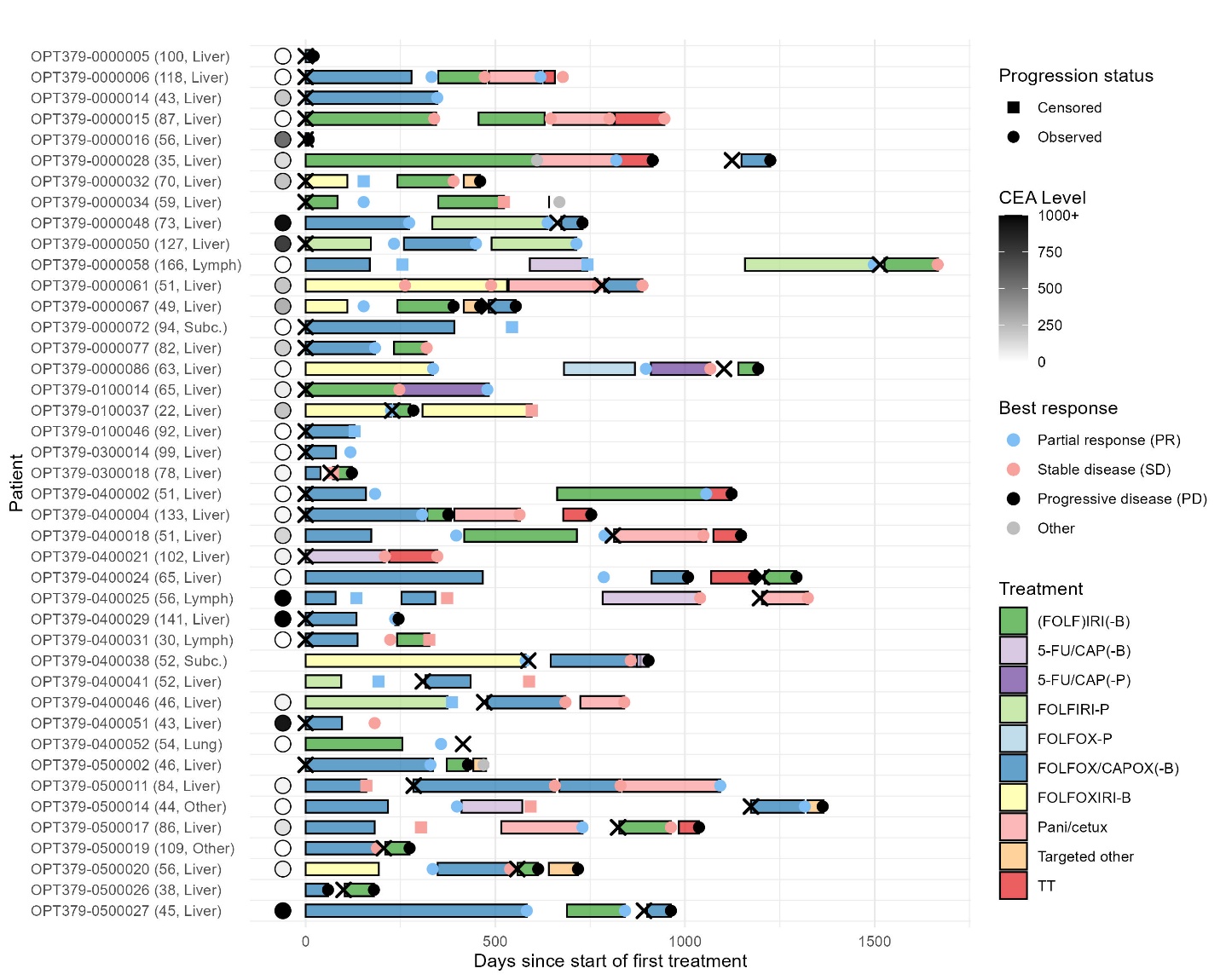


**Supplementary Figure S3.** Course of disease and treatments per patient. Horizontal bars represent treatment periods. Dots indicate the time of progression and squares indicate the time of censoring, colored by best RECIST response. Crosses mark biopsy dates, and colored circles show CEA levels in µg/L. Numbers next to patient IDs indicate days of expansion between biopsy and freezing for drug screen, with metastatic site of the biopsy. Time is in days since start of first treatment. Abbreviations: 5-FU (5-fluorouracil), B (bevacizumab), CEA (carcinoembryonic antigen), cetux (cetuximab), P/pani (panitumumab, TT (trifluridine/tipiracil).


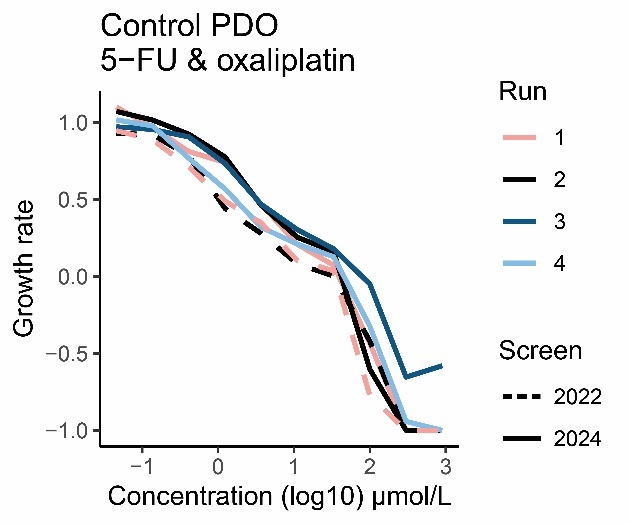


**Supplementary Figure S4.** Drug response curves of control PDO biological replicates screened for 5-FU & oxaliplatin in two biological replicates in 2022 and four biological replicates in 2024. Abbreviations: 5-FU (5-fluorouracil), PDO (patient-derived organoid).


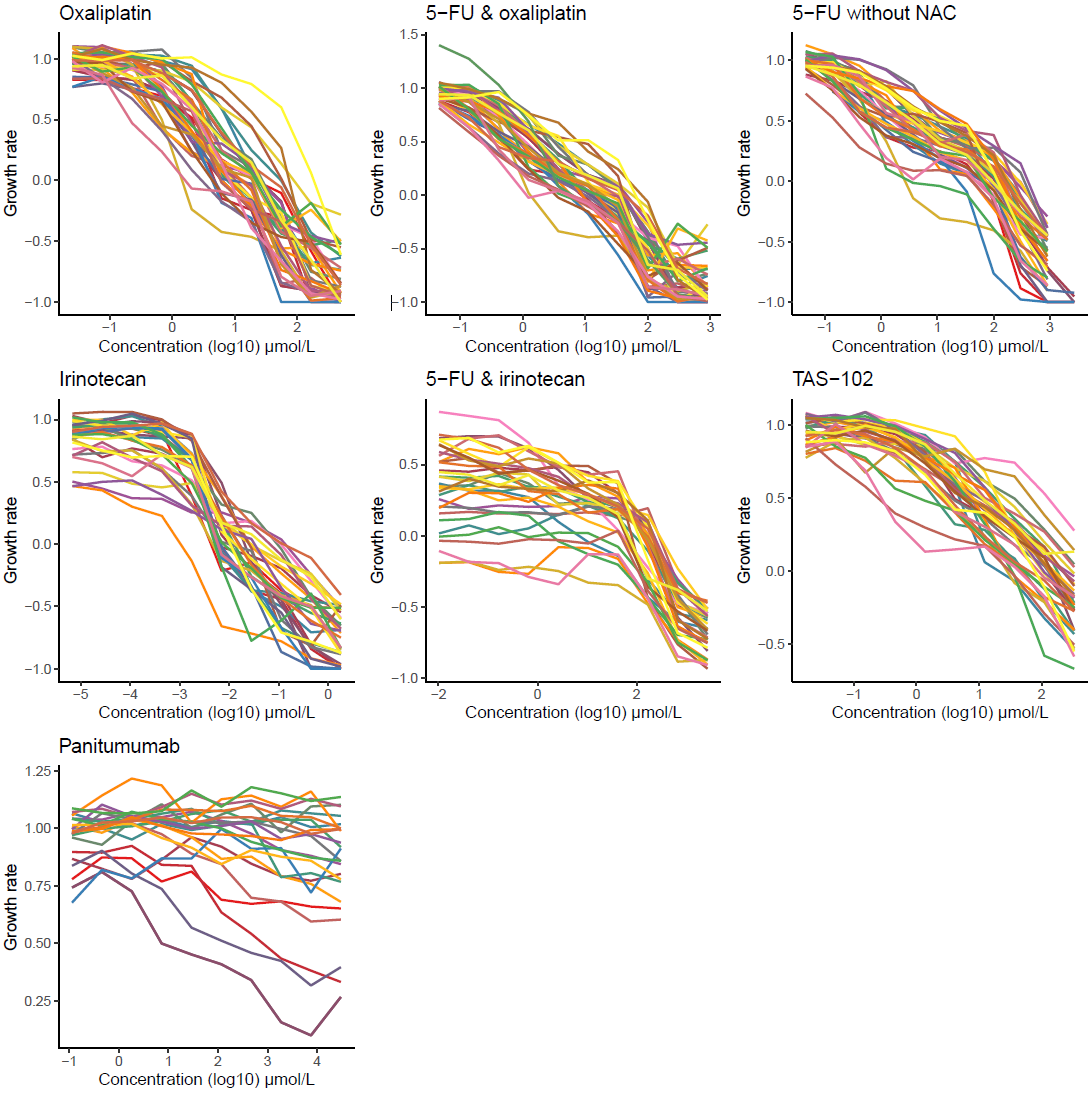

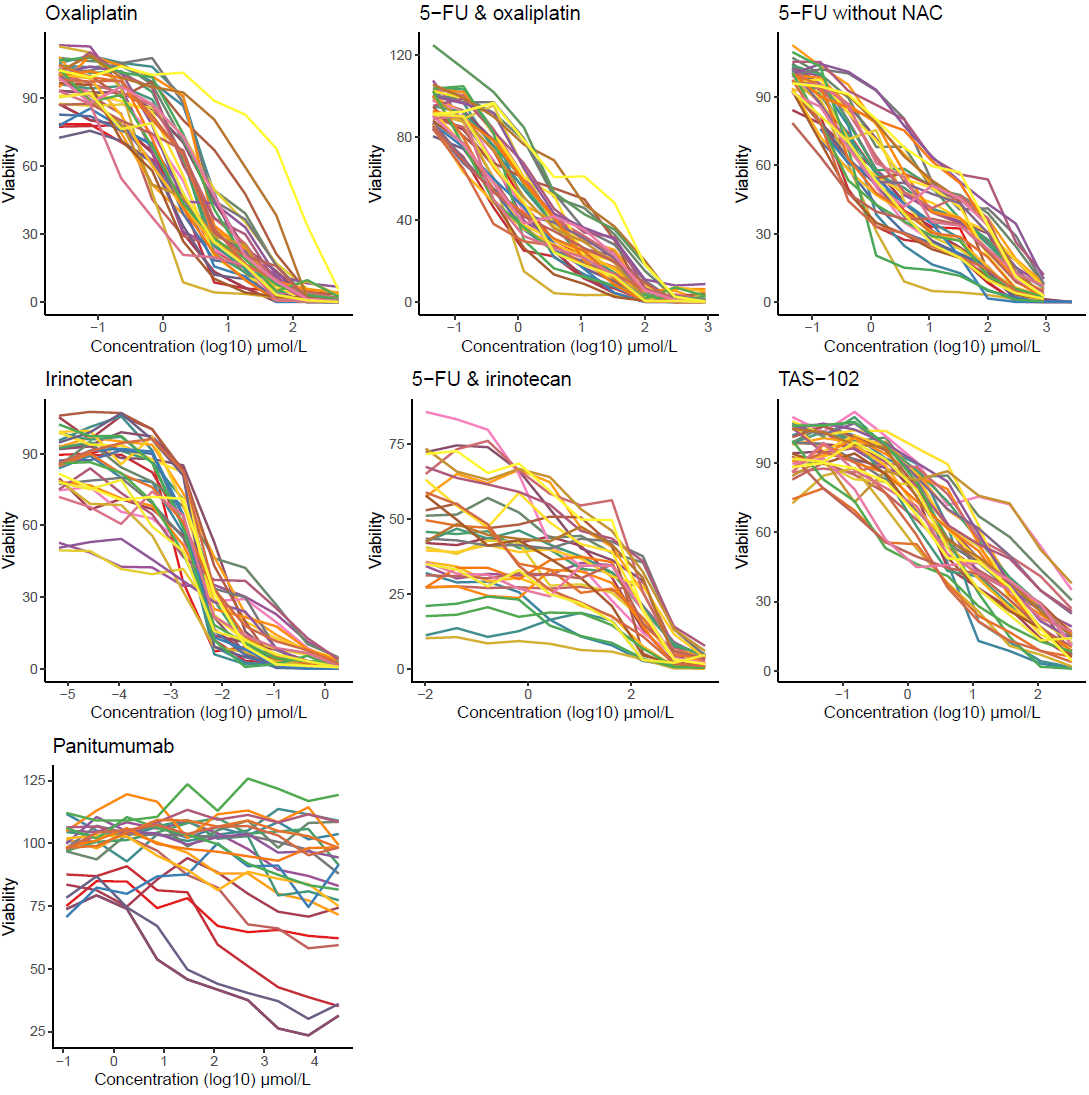


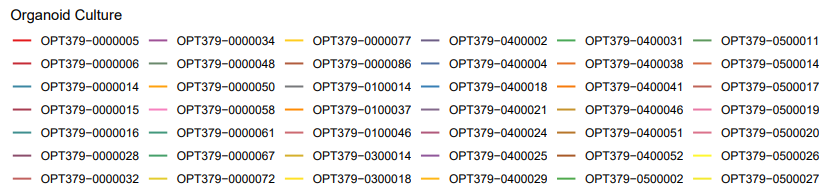


**Supplementary Figure S5.** Individual drug response curves for each patient-derived organoid per treatment. Abbreviations: 5-FU (5-fluorouracil), NAC (N-acetylcysteine).


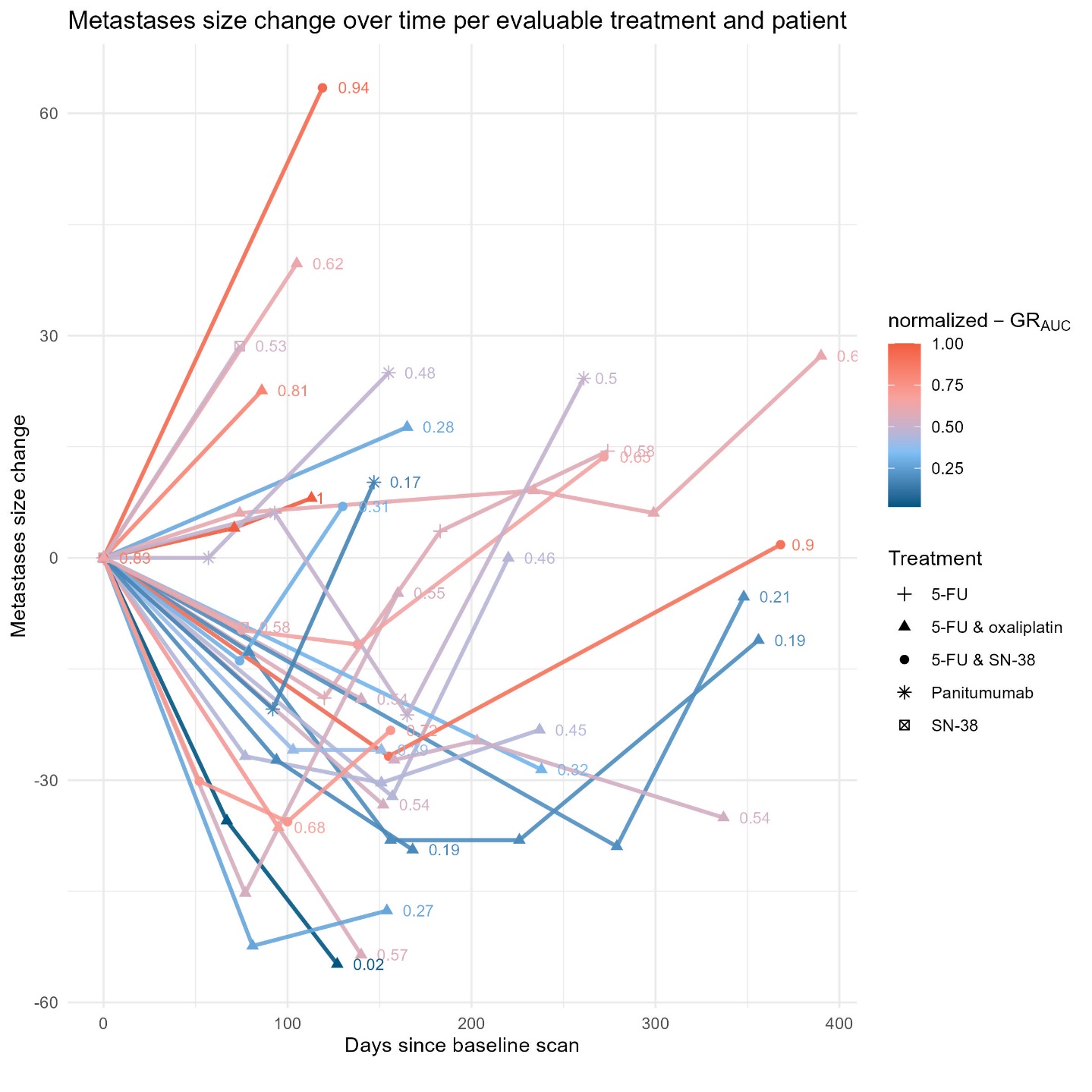


**Supplementary Figure S6.** Size change of all target lesions per patient and evaluable treatment over time is shown over sequential scans. A decrease in size on the last scan could indicate that, for example, treatment and scanning are stopped due to non-target progression or eligibility for resection, or the progression scan lacks size measurements and is not included in the plot. The color of the line indicates the organoid drug response (normalized GR_AUC_). Labels next to each line show the normalized GR_AUC_ value corresponding to that treatment. Abbreviations: AUC (area under the curve), 5-FU (5-fluorouracil), GR_AUC_ (area under the growth rate inhibition curve), SN-38 (active metabolite of irinotecan).

*
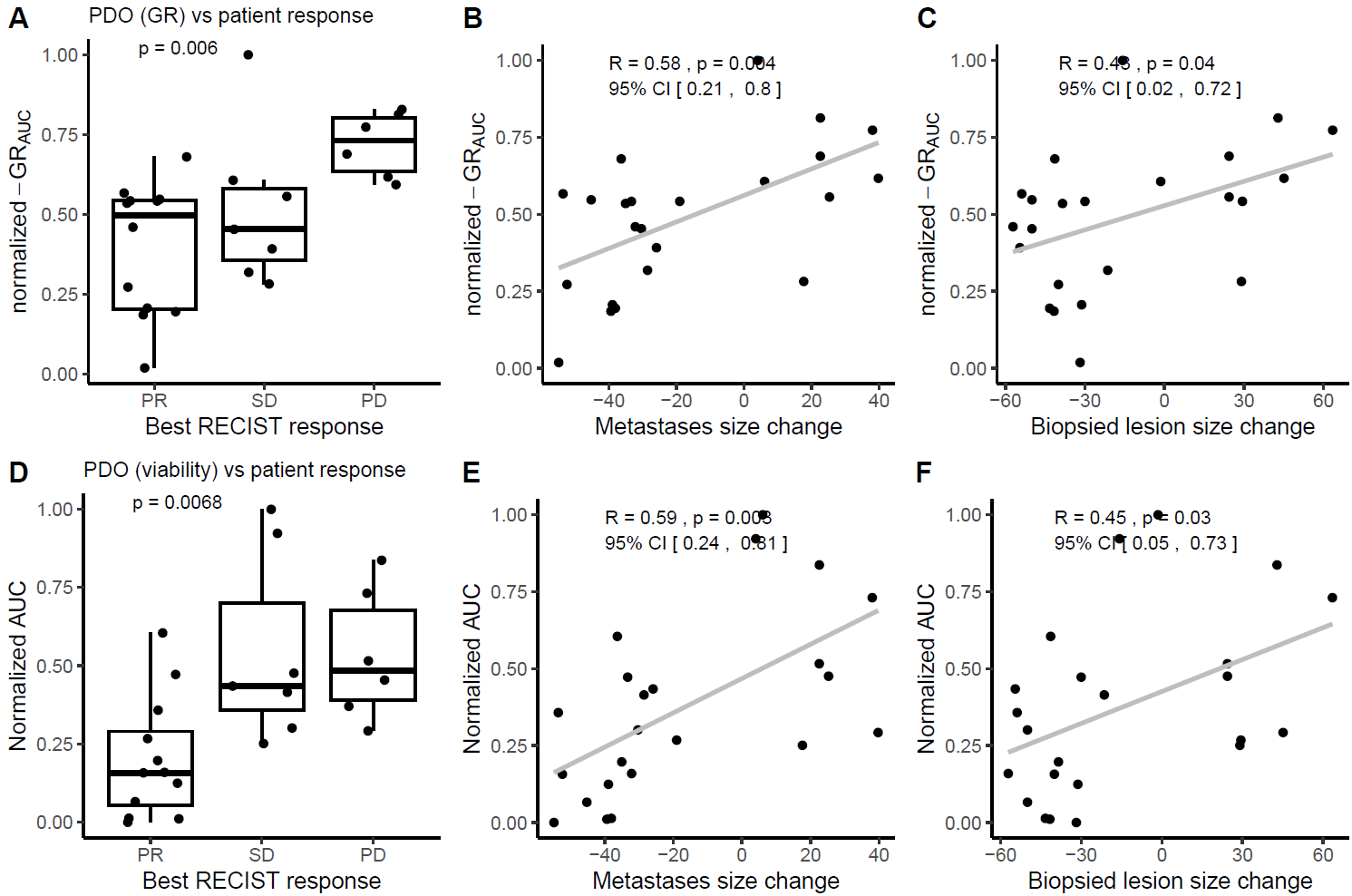
*

**Supplementary Figure S7.** Correlations between patient response (best RECIST response, size change of all target lesions, and the biopsied lesion) and organoid response (normalized GR_AUC_ in **A-C** and AUC in **D-F**) to 5-FU & oxaliplatin combination treatment. Abbreviations: 5-FU (5-fluorouracil), AUC (area under the curve), GR_AUC_ (area under the growth rate inhibition curve).


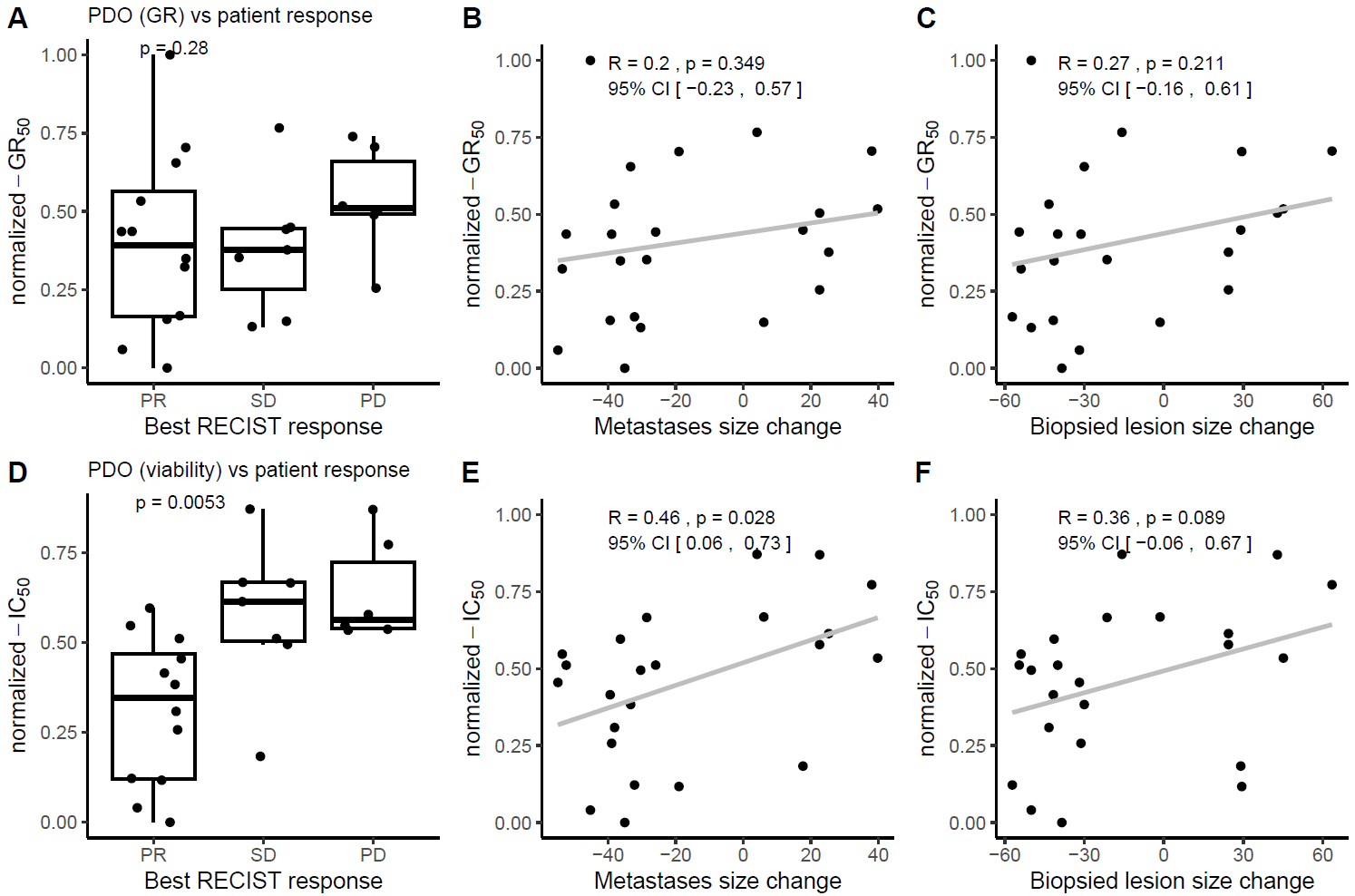


**Supplementary Figure S8.** Correlations between patient response (best RECIST response, size change of all target lesions, and the biopsied lesion) and organoid response (normalized IC_50_ in **A-C** and GR_50_ in **D-F**) to 5-FU & oxaliplatin combination treatment. Abbreviations: 5-FU (5-fluorouracil), AUC (area under the curve), GR_50_ (concentration that gives 50% growth rate inhibition), IC_50_ (concentration that gives 50% viability inhibition).
